# ncHMR detector: a computational framework to systematically reveal non-classical functions of histone modification regulators

**DOI:** 10.1186/s13059-020-01953-0

**Published:** 2020-02-24

**Authors:** Shengen Hu, Dawei Huo, Zhaowei Yu, Yujie Chen, Jing Liu, Lin Liu, Xudong Wu, Yong Zhang

**Affiliations:** 1 Institute for Regenerative Medicine, Shanghai East Hospital, Shanghai Key Laboratory of Signaling and Disease Research, Frontier Science Center for Stem Cell Research, School of Life Sciences and Technology, Tongji University, Shanghai, 200092 China; 2 Department of Cell Biology, Tianjin Medical University, 2011 Collaborative Innovation Center of Tianjin for Medical Epigenetics, Tianjin Key Laboratory of Medical Epigenetics, Qixiangtai Road 22, Tianjin, China; 3 Department of Neurosurgery, Tianjin Medical University General Hospital, Tianjin, China; 4 Present address: Key Laboratory of Forensic Genetics, National Engineering Laboratory for Forensic Science, Institute of Forensic Science, Beijing, China; 5 Department of Epidemiology, Harvard T.H. Chan School of Public Health, Boston, MA USA; 6 State Key Laboratory of Experimental Hematology, Institute of Hematology and Blood Diseases Hospital, Chinese Academy of Medical Sciences and Peking Union Medical College, Tianjin, 300020 China

**Keywords:** Histone modification regulator, Non-classical function, Computational framework, Chromatin regulation

## Abstract

Recently, several non-classical functions of histone modification regulators (HMRs), independent of their known histone modification substrates and products, have been reported to be essential for specific cellular processes. However, there is no framework designed for identifying such functions systematically. Here, we develop ncHMR detector, the first computational framework to predict non-classical functions and cofactors of a given HMR, based on ChIP-seq data mining. We apply ncHMR detector in ChIP-seq data-rich cell types and predict non-classical functions of HMRs. Finally, we experimentally reveal that the predicted non-classical function of CBX7 is biologically significant for the maintenance of pluripotency.

## Background

**Fig. 1:**
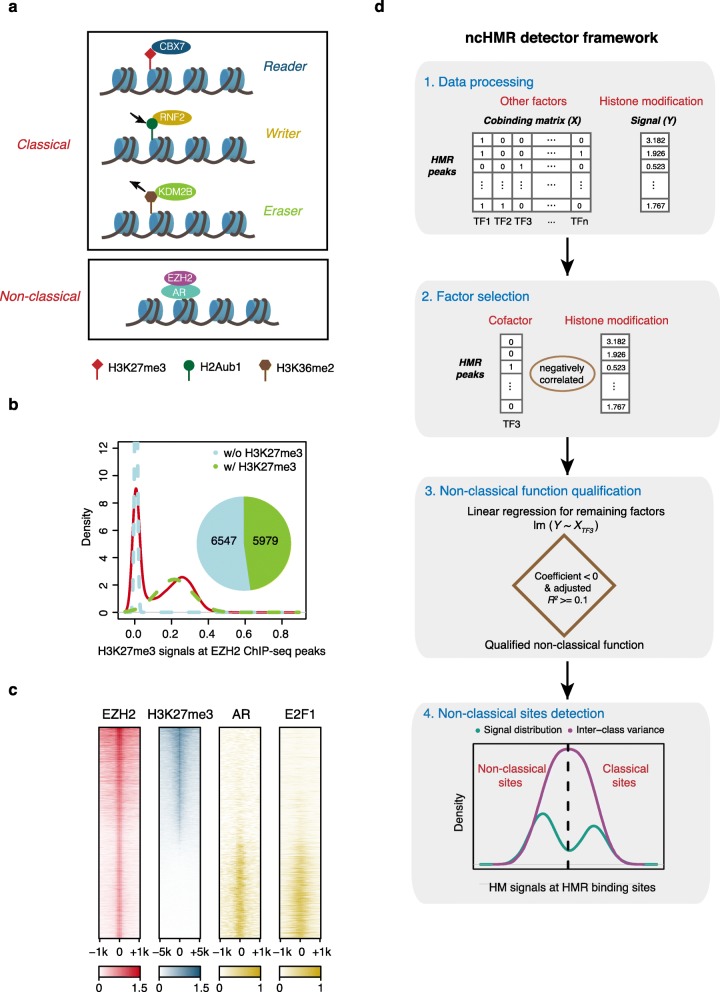
Non-classical functions of HMRs and ncHMR detector framework. **a** Schematic showing classical and non-classical functions of HMRs. Classical functions of HMRs include recognizing (CBX7 recognizes H3K27me3), adding (RNF2 catalyzes H2Aub1), or removing (KDM2B demethylates H3K36me2) histone modification substrates/products. In contrast, non-classical functions of HMRs are independent of its classical histone modification substrates/products and some involve in cooperation with other cofactors (EZH2 interacts with AR to activate gene transcription independently of H3K27me3). **b** Graph showing the classical and non-classical binding sites of EZH2 in abl cell line. The red line indicates the distribution of H3K27me3 signals at EZH2 ChIP-seq peaks. The light blue and green dashed lines indicate two fitted normal distributions for H3K27me3 signals which represent non-classical sites without H3K27me3 and classical sites with H3K27me3, respectively. The pie chart shows the number of two kinds of binding sites. **c** Heatmap showing EZH2, H3K27me3, and E2F1 enrichment around EZH2 ChIP-seq peak centers in abl cell line. Rows represent EZH2 binding sites and are ranked by normalized H3K27me3 signals. The colors indicate the normalized ChIP-seq enrichment level and the values are scaled by row. **d** A schematic view of the workflow of the ncHMR detector framework (see the “Methods” section for details). All ChIP-seq data used in the analysis were annotated in Additional file 1: Fig. S1a

Histone modification regulators (HMRs) are proteins that can recognize, add, or remove modifications on histone tails [1, 2], usually termed as histone modification (HM) readers, writers, or erasers, respectively (Fig. 1a). Numerous studies have shown that perturbing HMRs can lead to various diseases, and some HMRs are potential therapeutic targets [3–5], demonstrating their critical roles in regulating chromatin state and gene expression. In addition to their classical functions as HM readers, writers, or erasers, some HMRs have been reported to perform non-classical regulatory functions in chromatin, which are independent of their known HM substrates/products (Additional file 1: Fig. S1a), in a context-dependent manner by cooperating with cofactors. For example, histone methyltransferase EZH2, a core unit of PRC2 complex, can play a PRC2-independent role by interacting with androgen receptor (AR) to activate a subset of its target genes in an androgen-independent prostate cancer cell line [6] (Fig. 1a). In another example, SETDB1, a histone methyltransferase responsible for the methylation of histone H3 lysine 9 (H3K9) [7], can modulate PRC2 activity at developmental genes independently of H3K9me3 in mouse embryonic stem cells (mESCs) [8]. These emerging cases suggest that the non-classical functions of HMRs can be essential to certain cellular processes.

Various technologies have been applied to discover the non-classical regulatory functions of HMRs. For example, streptavidin bead complex isolation followed by mass spectrometric analysis approach identified the non-classical function of RNF2, a key unit of PRC1, through its interaction with KDM1A [9]. In another example, by using sequential ChIP and ChIP-qPCR technologies, KDM4B, a demethylase of H3K9me3 or H3K36me3, was found to interact with MLL2 complex, an H3K4-specific methyltransferase, to regulate the breast carcinogenesis gene [10]. In addition to the above low-throughput technologies, analyzing ChIP-seq data can also contribute to the discovery of non-classical functions of HMRs by focusing on those binding sites of HMRs without signals of their classical substrates or products; 11 out of 18 known cases were discovered by this approach (Additional file 1: Fig. S1a). ChIP-seq technology has been widely used to profile the genome-wide binding sites of transcription factors (TFs), chromatin regulators, and HMs [11], providing a valuable resource for the efficient identification of non-classical functions of HMRs.

In the last decade, many computational approaches have been developed to perform in-depth analysis on ChIP-seq data of HMs and TFs, but none of them was designed to predict non-classical functions of HMRs. HMCan [12] and ChIPseqR [13] were specially designed to accurately identify genomic loci of HMs, while Epigram [14] and DeepHistone [15] can be used to predict loci of HMs based on sequence features and chromatin accessibility. DeepChrome [16], EpiRegNet [17], and Epidaurus [18] were designed for revealing the regulatory functions of HMs on transcription regulation. However, the aims of the above methods were fundamentally different to the identification of non-classical functions of HMRs. MultiGPS [19], edgeR [20], and DBChIP [21] can be applied to identify the condition-specific binding sites of a given HMR among multiple conditions, which were related, but distinct to identify its context-dependent binding sites in a given condition. Gerstein et al. [22] developed the factor co-association analysis method, which could be applied to identify cofactors at specific subsets of a given HMR’s binding sites. However, it cannot distinguish cofactors of classical and non-classical functions of a given HMR. To the best of our knowledge, there is no systematic computational framework designed for identifying the non-classical functions of HMRs based on ChIP-seq data integration, mainly due to the following challenges. First, a typical HMR ChIP-seq dataset has thousands of peaks or more, and experimental variation could result in the non-specific missing of its classical substrate or product signals on a fraction of peaks, which may in turn lead to a high false discovery rate in non-classical function prediction. Second, the quality of public ChIP-seq data is highly variable, and stringent quality control (QC) is necessary to guarantee the reliability of prediction. Therefore, to take advantage of public ChIP-seq data in detecting non-classical functions of HMRs, novel computational frameworks are needed to solve the above challenges.

In this study, we presented ncHMR detector (non-classical functions of histone modification regulator detector), a computational framework for predicting non-classical functions of HMRs and their cooperating cofactor candidates. This framework was designed to overcome the above challenges as follows. First, ncHMR detector includes a feature selection component, which is based on the significantly enriched co-occurrence of binding events of cofactors and the absence of classical substrates/products of a given HMR. The feature selection step can help to largely avoid the influence of the presence or absence of non-specific signals in single ChIP-seq data. Second, we used a stringent QC criterion to filter the public ChIP-seq data to guarantee the quality of datasets used in the prediction framework. In addition to the prediction of non-classical functions and cofactors of HMRs, ncHMR detector can also report the genomic loci with predicted non-classical functions through Otsu’s method, an image processing algorithm [23]. We applied ncHMR detector to ChIP-seq data-rich cell types, including GM12878, K562, hESCs, mESCs, HeLa, and HepG2, and predicted 12 non-classical functions of HMRs and their cofactor candidates. To confirm the accuracy of the prediction, we experimentally validated the predicted non-classical function of CBX7, a component of PRC1 complex [24], in mESCs. Our results showed that the H3K27me3-independent non-classical function of CBX7 is closely related to the pluripotency of mESCs, with NANOG, a key effector regulating the pluripotency [25], as the cofactor. The source code of ncHMR detector is available in https://github.com/TongjiZhanglab/ncHMR_detector.

## Results

### ncHMR detector framework

To identify the ChIP-seq data features of non-classical functions of HMRs, we collected previously reported non-classical functions of HMRs (Additional file 1: Fig. S1a) and reanalyzed the 11 cases discovered by mining ChIP-seq data. In all 11 reported cases, the lack of classical substrate/product signals at 50% or more of ChIP-seq peaks is common for HMRs with reported non-classical functions, and the overlap percentages between ChIP-seq peaks of such HMRs and their classical substrates/products are much lower than those randomly selected HMR ChIP-seq datasets (Additional file 1: Fig. S1b). For example, consistent with the previous report, we observed a bimodal distribution of the H3K27me3 signal at EZH2 ChIP-seq peaks in LNCaP-abl (abl) cell line, an androgen-independent prostate cancer cell line (Fig. 1b), where the two modes represent classical and non-classical binding sites of EZH2, respectively. Furthermore, among the 11 cases, 7 of them have reported cofactors that interact with HMRs and are required for non-classical functions of HMRs (Additional file 1: Fig. S1a). We profiled the ChIP-seq signals of reported cofactors at ChIP-seq peaks of HMRs and found that 6 reported cofactors showed co-localization at non-classical binding sites of HMRs with low classical substrate/product signals (Fig. 1c, Additional file 1: Fig. S1c-f). For example, AR and E2F1 are two cofactors reported to cooperate with EZH2 to perform a non-classical function in abl [6, 26], and their ChIP-seq signals were enriched at non-classical sites of EZH2 (Fig. 1c, Additional file 1: Fig. S1g). Taken together, the binding sites of HMRs with reported non-classical functions exhibited the enriched co-occurrence of binding events of cofactors and the absence of classical substrates/products. The above observations motivated us to pursue identifying more non-classical functions of HMRs through ChIP-seq data mining.

Based on the ChIP-seq data features of reported non-classical functions of HMRs, we designed ncHMR detector, a computational framework to systematically predict the non-classical functions and cofactors of a given HMR. The framework relies on the significantly enriched co-occurrence of cofactor binding events and the absence of classical substrates/products of each given HMR. It includes four steps (Fig. 1d, see the “Methods” section for details). In the first step, public ChIP-seq data of the given HMR, its classical HM substrates/products, and TFs from the same cell type were collected and filtered based on certain QC criterion (see the “Methods” section for detail). The design matrix *X* was generated to represent the cobinding occurrence (0 or 1) of other factors (including TFs and other HMRs) at each ChIP-seq peak of the given HMR. The average HM signals around each ChIP-seq peak center of the given HMR were stored in a response vector *Y*. We used ± 5 kb flanking peak centers to calculate the average signals for well-known broad HMs, including H3K9me3, H3K27me3, and H3K36me3, while used ± 1 kb for other HMs. In the second step, to avoid the confounding influence of too many cofactor predictors (i.e., the TFs and other HMRs with ChIP-seq data in the same cell type), a feature selection method based on penalized linear regression [27] (either elastic [28] or Lasso regression [29]) was applied to only keep the negative correlated factors in *X* in predicting the HM signals in *Y*. In the third step, for each of the remaining factors after feature selection, a univariate linear regression was refitted between the cobinding occurrence of the factor and vector *Y*. If one or more factors showed strong negative correlations, the given HMR was regarded as having a potential non-classical function, and those negatively correlated factors were regarded as cofactor candidates. In the fourth step, to report the genomic loci with predicted non-classical functions, the ChIP-seq peaks of the given HMR were classified into classical and non-classical binding sites by using Otsu’s method, an image processing algorithm [23]. Considering that an HMR may cooperate with multiple cofactors independently, the framework reported subsets of the non-classical sites overlapping with the binding sites of each cofactor candidate.

### Performance evaluation of ncHMR detector

The non-specific absence of a HMR’s classical substrate/product signals on a fraction of its ChIP-seq peaks due to experimental variation may cause the non-classical function prediction to exhibit low specificity or robustness (prediction sensitive to noise). To evaluate the prediction performance of ncHMR detector, we designed two different setups for specificity and robustness evaluation based on simulated data. The first setup is designed to evaluate the specificity of cofactor identification. Based on EZH2 and H3K27me3 ChIP-seq data in mESC, we simulated the cobinding events with EZH2 for four groups of other factors, and different groups displayed distinct correlations (strong negative, weak negative, weak positive, and strong positive) between cobinding occurrence with EZH2 (0 or 1) and the response H3K27me3 signals (see the “Methods” section for details). Two other feature selection methods, greedy forward selection [30] and knockoff [31], were also used in the evaluation. Based on the evaluation dataset, classifying the factors in the strong negative group as true positive cofactors and other factors as true negative, ncHMR detector with elastic net or Lasso as the feature selection method showed high specificity (0.98 ± 0.03 for elastic net and 0.98 ± 0.03 for Lasso), which are much larger than the specificities obtained by greedy forward selection (0.83± 0.03) and knockoff (0.92 ± 0.03). Furthermore, the predicted cofactors showed a higher frequency of cobinding events with EZH2 at non-classical sites (Additional file 1: Fig. S2a), confirming the high specificity of cofactor identification using ncHMR detector.

**Fig. 2:**
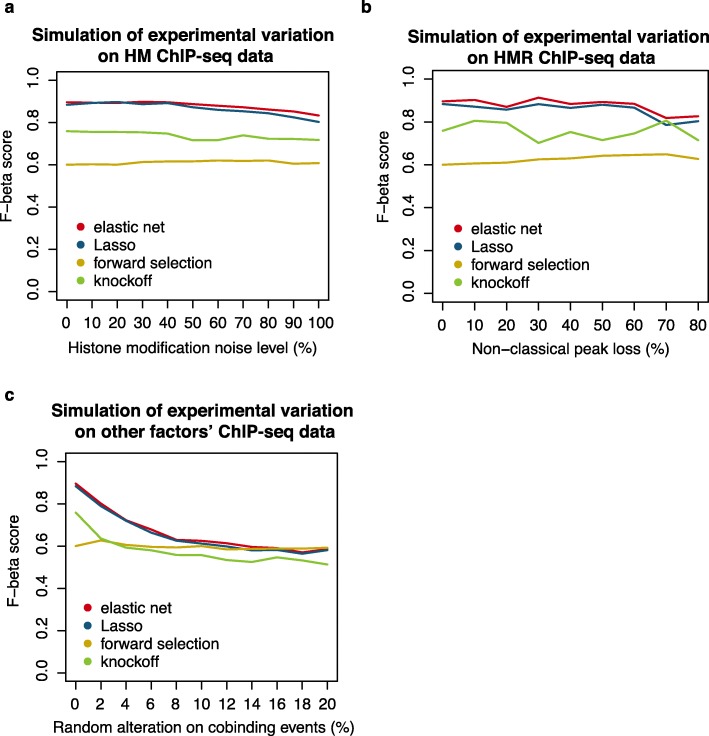
Performance evaluation of ncHMR detector. **a**–**c** Graph showing robustness of cofactor identification on evaluation data with three types of noise that simulate three types of experimental variation. **a** A Gaussian noise on the H3K27me3 signal at EZH2 ChIP-seq peaks, the mean and standard deviation of the noise distribution were set to be equal to a given fraction (from 0 to 100%) of the average histone modification signal across the genome. **b** Random loss of a fraction (from 0 to 80%) of non-classical sites of EZH2. **c** Random alteration on a fraction (from 0 to 20%) of cobinding events with EZH2 for other factors. The F-beta score (β = 0.75) was used to evaluate the robustness by treating the factors in the strong negative group as true positive cofactors and other factors as true negative. The red, blue, yellow, and green lines represent cofactors identified by elastic net, Lasso, forward selection, and knockoff, respectively

The second setup is designed to evaluate the robustness of cofactor identification. Three types of noises were added to the evaluation dataset, including (1) adding a Gaussian noise on H3K27me3 signal at EZH2 ChIP-seq peaks to simulate the experimental variation on HM ChIP-seq data, (2) randomly losing a fraction of non-classical sites of EZH2 to simulate the experimental variation on HMR ChIP-seq data, and (3) randomly altering the cobinding events with EZH2 for other factors to simulate the experimental variation on other factors’ ChIP-seq data (see the “Methods” section for details). We used the F-beta score and specificity to evaluate the robustness of cofactor identification in each simulation condition, and ncHMR detector with elastic net or Lasso as the feature selection method showed high F-beta score and specificity upon all three types of noise, which are higher than those obtained using greedy forward selection or knockoff (Fig. 2a–c, Additional file 1: Fig. S2b-d), confirming the high robustness of cofactor identification using ncHMR detector.

Although there is no existing computational framework designed for identifying the non-classical functions of HMRs based on ChIP-seq data integration, some methods can be modified to perform the prediction (see the “Methods” section for details). We compared the performance of ncHMR detector (elastic net and Lasso) and four modified existing methods (MultiGPS + Jaccard index, edgeR + Jaccard index, DBChIP + Jaccard index, modified factor co-association analysis) by evaluating the robustness of cofactor identification (see the “Methods” section for details), and ncHMR detector showed much higher F-beta scores than those four modified existing methods (Additional file 1: Fig. S3a, b). Considering Gerstein et al. applied factor co-association analysis by using normalized peak intensities (quantitative values, ranging from 0 to 1) rather than the presence or absence of cobinding events (binary values, 0 or 1) in the cobinding matrix [22], we also compared the performance of ncHMR detector and modified factor co-association analysis based on simulated cobinding matrix with quantitative values (ranging from 0 to 1) (see the “Methods” section for details), and ncHMR detector showed much better performance than modified co-association analysis (Additional file 1: Fig. S3c, d). Taken together, ncHMR detector outperformed those existing methods which can be modified to identify the non-classical functions of HMRs.

### Prediction of non-classical functions in ChIP-seq data-rich cell types

**Fig. 3:**
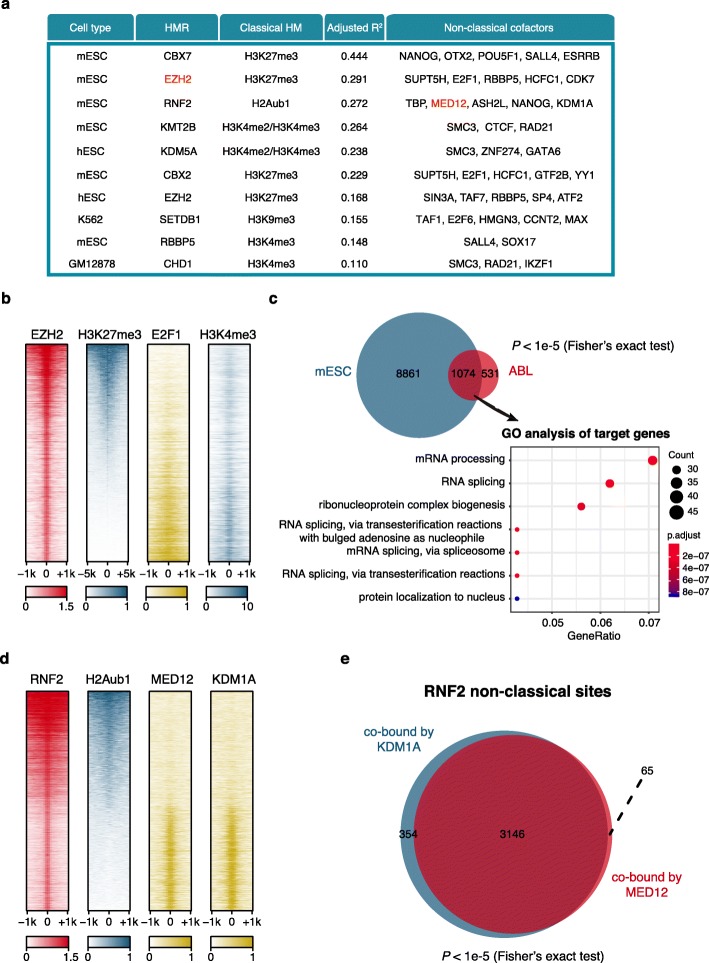
Prediction of non-classical functions in ChIP-seq data-rich cell types. **a** Top 10 ranked non-classical functions and the corresponding cofactor candidates of HMRs. Non-classical functions of HMRs are ranked by adjusted *R*^*2*^ of the cofactor. For non-classical functions candidates from multiple ChIP-seq data of the same HMR, the top ranked non-classical function candidates are kept. For each non-classical function candidate, the top 5 cofactor candidates are showed. The previously reported non-classical functions are highlighted in red. **b** Heatmap showing EZH2, H3K27me3, E2F1, and H3K4me3 enrichment around EZH2 ChIP-seq peak centers. Rows represent EZH2 binding sites and are ranked by the normalized H3K27me3 signals at EZH2 binding sites. The colors indicate the normalized ChIP-seq enrichment level and the values are scaled by row. EZH2, E2F1, H3K27me3, and H3K4me3 ChIP-seq data in mESCs were obtained from GSE49431, GSE11431, GSE58023, and GSE73432. **c** Venn diagram showing the significant overlap of target promoters (±3 kb around TSSs of genes) between EZH2 non-classical sites cobound by E2F1 in mESCs and converted EZH2 non-classical sites cobound by E2F1 from human abl cell line. Fisher’s exact test was performed to identify statistical significance. The dot plot shows that target genes of overlap sites were enriched in biological processes such as mRNA processing. Gene ontology analysis of target genes was performed using the R package clusterProfiler [34]. Top 7 significant (Benjamini-Hochberg-adjusted *p* value < 0.01) terms are shown. **d** Heatmap showing RNF2, H2Aub1, MED12, and KDM1A enrichment around RNF2 ChIP-seq peak centers. Rows represent RNF2 binding sites and are ranked by the normalized H2Aub1 signals at RNF2 binding sites. The colors indicate the normalized ChIP-seq enrichment level and the values are scaled by row. RNF2, MED12, KDM1A, and H2Aub1 ChIP-seq data were obtained from GSE55697, GSE22557, GSE27841, and GSE34518. **e** Venn diagram showing the significant overlap between non-classical RNF2 sites cobound by MED12 and sites cobound by KDM1A. Fisher’s exact test was performed to identify statistical significance

As ncHMR detector relies on the significantly enriched co-occurrence of binding events of cofactors and the absence of classical substrates/products of the given HMR, the availability of a large amount of ChIP-seq data for other factors in the same cell type is required for effective cofactor identification. In this study, we applied ncHMR detector in four ChIP-seq data-rich cell types, including GM12878, K562, hESCs, mESCs, HeLa, and HepG2 (Additional file 1: Fig. S4a). In total, 12 non-classical functions of HMRs, together with cofactor candidates, were predicted by ncHMR detector (Additional file 2: Table S1). Among the top 10 ranked non-classical function candidates of HMRs, 2 cases have been partially reported, in terms of either as the HMR having a non-classical function (for example, EZH2 has non-classical function in mESCs [32]) or the HMR having predicted cofactor (for example, RNF2 interacts with MED12 in mESCs [33]) (Fig. 3a).

EZH2 was predicted to have a non-classical function in mESCs, which is consistent with a previous study [32]. However, to the best of our knowledge, whether EZH2 functions with any cofactors at non-classical binding sites in mESCs is still unexplored. In this study, ncHMR detector predicted several cofactor candidates that may function with EZH2 at its non-classical binding sites in mESCs, including SUPT5H, E2F1, HCFC1, CDK7, and RBBP5. Among the predicted cofactor candidates, E2F1 was reported as the cofactor of EZH2’s non-classical function in abl cell line [26], indicating that it may also function as a cofactor of EZH2 to activate target genes in mESCs. ChIP-seq signal profiles of EZH2, H3K27me3, and E2F1 in mESCs confirmed the co-occurrence of EZH2 and E2F1 at genomic loci without H3K27me3 signals but instead with strong H3K4me3 signals (Fig. 3b, Additional file 1: Fig. S4b). It was reported that the cooperation of EZH2 and E2F1 in transcriptional activation is conserved in diffuse large B cell lymphomas [26], which inspired us to investigate whether such cooperation is conserved across species. We converted the genomic coordinates of EZH2 non-classical sites cobound by E2F1 in abl to the mouse genome, target promoters of those sites were significantly overlapped with the counterpart in mESCs, and genes associated with the overlapping EZH2 non-classical sites were enriched in biological processes such as mRNA processing (Fig. 3c). It suggests that the non-classical function of EZH2 in cooperation with E2F1 could be conserved across different cell types and species. RNF2, a key unit of the PRC1 complex, catalyzes the mono-ubiquitylation of histone H2A on lysine 119 (H2AK119ub1) [35] and has been reported to interact with MED12 in mESCs [33]. However, whether such an interaction occurs independently of RNF2’s classical function is still unexplored. In this study, RNF2 was predicted to have a non-classical function in mESCs, with MED12 as one of the cofactor candidates. In addition, among the predicted cofactor candidates, KDM1A was reported to interact with RNF2 in erythroleukemia cells [9], indicating that it may also function as a cofactor of RNF2 in mESCs. ChIP-seq signal profiles of RNF2, H2AK119ub1, MED12, and KDM1A in mESCs confirmed the co-occurrence of three factors at genomic loci without H2AK119ub1 signals (Fig. 3d, Additional file 1: Fig. S4c). Furthermore, RNF2 non-classical sites cobound by MED12 are significantly overlapped with those cobound by KDM1A (Fig. 3e), suggesting that RNF2, MED12, and KDM1A may function together in mESCs. The analysis of both partially reported cases indicated that the ncHMR detector prediction not only can indicate the existence of non-classical function for a given HMR, but also provide valuable information for the investigation of its mechanism.

It is possible that some HMRs’ non-classical functions may be correlated with their classical functions. To investigate that possibility, for each predicted HMR with non-classical function, we calculated the average distance between non-classical sites and their nearest classical sites and compared it with the average nearest distance within non-classical sites. Among 12 predicted HMRs with non-classical functions, KDM5A, RBBP5, and WDR5 showed significantly closer distance between their non-classical sites and classical sites (Additional file 1: Fig. S4d). We further investigated their chromatin interaction frequencies using public Hi-C data, and we observed significantly higher interaction frequencies between non-classical sites and their nearest classical sites for KDM5A, RBBP5, and WDR5 (Additional file 1: Fig. S4e). Those results suggested that the non-classical functions of KDM5A, RBBP5, and WDR5 might be correlated with their classical functions via chromatin looping.

### Non-classical function of CBX7 for the maintenance of pluripotency

**Fig. 4:**
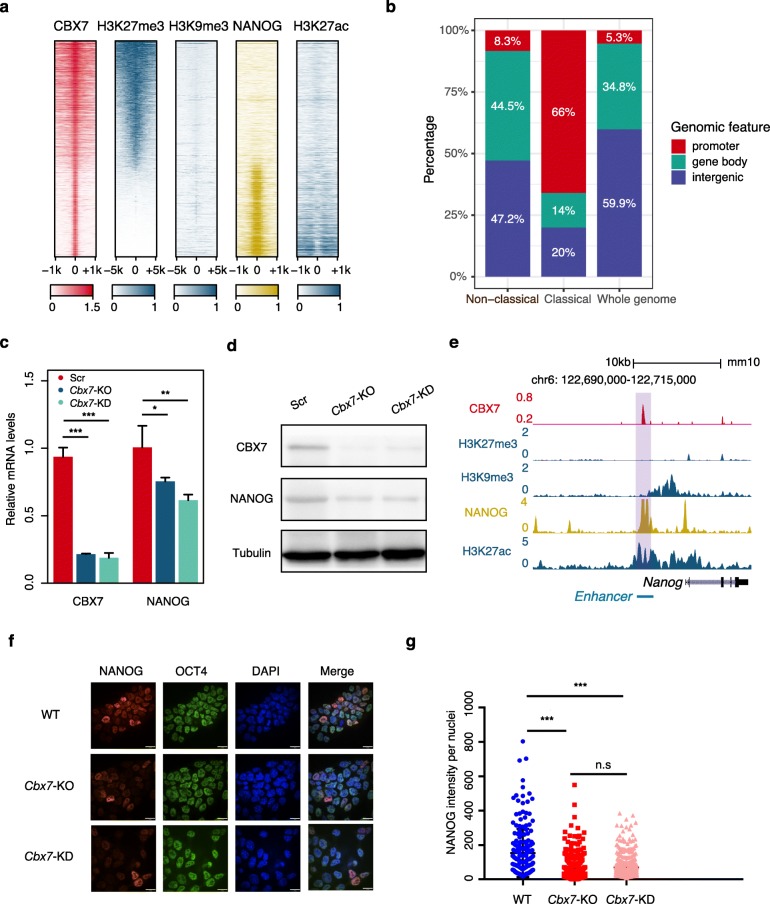
Non-classical function of CBX7 for the maintenance of pluripotency. **a** Heatmap showing CBX7, CBX7’s classical substrates H3K27me3 and H3K9me3, predicted cofactor NANOG, and H3K27ac enrichment around CBX7 ChIP-seq peak centers. Rows represent CBX7 binding sites and are ranked by normalized H3K27me3 signals. The colors indicate the normalized ChIP-seq enrichment level and the values are scaled by row. CBX7, NANOG, H3K27me3, H3K9me3, and H3K27ac ChIP-seq data were obtained from GSE64008, GSE90893, GSE58023, GSE90895, and GSE67867. **b** Stacked bar plot showing the percentages of classical sites, non-classical sites, and the whole genome that reside in promoter, gene body, and intergenic regions. The promoter is defined as ± 3 kb around TSS of the gene. **c** Real-time qPCR analysis for the expression of CBX7 and NANOG in wild type, *Cbx7*-knockout, and *Cbx7*-knockdown mESCs. Error bars represent the standard deviation for triplicate experiments, and unpaired *t* test with Welch’s correction was used to calculate the statistical significance for comparison (*p value < 0.05, **p value < 0.01, ***p value < 0.001, and *n.s* represents non-significant). **d** Western blot analysis of CBX7 and NANOG level in wild type, *Cbx7*-knockout, and *Cbx7*-knockdown mESCs. Tubulin was used as a loading control. Blots were cut before antibody application. Gel images for Western blot are shown in Additional file 1: Fig. S6a. **e** The UCSC genome browser view of CBX7, H3K27me3, H3K9me3, NANOG, and H3K27ac enrichment at a previously reported enhancer of *Nanog* [37]. Enhancer loci are shaded in purple and signals represent ChIP-seq RPM. **f** Immunofluorescence staining for NANOG (red), OCT4 (green), DAPI (blue), and merged images in wild type, *Cbx7*-knockout, and *Cbx7*-knockdown mESCs. **g** The fluorescence intensity of NANOG for about 300 nuclei in wild type, *Cbx7*-knockout, and *Cbx7*-knockdown mESCs. Unpaired *t* test with Welch’s correction was used to calculate statistical significance for comparison

In addition to partially reported non-classical functions, ncHMR detector also predicted 12 non-classical functions of HMRs, among which the non-classical function of CBX7, a component of the PRC1 complex that preferentially recognizes H3K27me3 and H3K9me3 by its CHRromatin Organization Modifier (CHROMO) domain [24], in mESCs ranked as the top prediction (Fig. 3a). NANOG, a well-known pluripotency factor [25], was predicted as the top cofactor candidate. The ChIP-seq signal profiles of CBX7, H3K27me3, H3K9me3, and NANOG in mESCs confirmed the co-occurrence of CBX7 and NANOG genomic loci without H3K27me3 or H3K9me3 signals but instead with strong H3K27ac signals, which is considered to be the mark of active promoters or enhancers in mammalian cells [36] (Fig. 4a, Additional file 1: Fig. S5a, b). Notably, the predicted CBX7 non-classical binding sites cobound by NANOG were far from transcription start sites (TSSs), while the classical binding sites were mainly localized at promoters (Fig. 4b). These data indicate that CBX7 may play a non-classical function in cooperation with NANOG in mESCs.

To rule out the possibility that CBX7 ChIP-seq signals at non-classical binding sites might be due to the non-specificity of antibody, we next examined whether the depletion of *Cbx7* can affect its binding at those sites. We depleted *Cbx7* in mESCs through either knockout or knockdown, and the expression levels of CBX7 were significantly downregulated (Fig. 4c, d; Additional file 1: Fig. S6a). The depletion of *Cbx7* by knockdown led to a dramatic decrease in enrichment levels at its non-classical binding sites (*Id3* enhancer and *Nanog* enhancer) as well as at classical binding sites (*Fgf3* promoter and *Wnt1* promoter), while as a negative control, the non-binding site (*Fgf4* promotor) remained unbound by CBX7 (Additional file 1: Fig. S5c). As CBX7’s non-classical binding sites had strong H3K27ac signals, we next investigated whether the H3K27ac signal is required for CBX7 binding. As bromodomains are known as the reader of lysine acetylation [38], we treated mESCs with JQ1, a selective inhibitor of the BET family of bromodomain proteins. The treatment of JQ1 indeed led to a dramatically decreased enrichment levels on CBX7’s non-classical binding sites, but not at its classical binding sites (Additional file 1: Fig. S5c), suggesting that CBX7 binding to its non-classical binding sites is dependent on a bromodomain-containing cofactor, which is completely different its CHROMO domain-dependent mechanism at the classical binding sites.

Among the predicted non-classical binding sites of CBX7, we observed the co-occurrence of CBX7 and NANOG at the distal upstream region of *Nanog*, which was reported as an enhancer of *Nanog* [37] (Fig. 4e). This finding prompted us to investigate whether CBX7 contributes to the formation of a positive auto-regulatory loop of NANOG expression, which is essential for the maintenance of naïve pluripotency [39]. Upon the efficient depletion of *Cbx7*, the expression level of NANOG is modestly but significantly downregulated (Fig. 4c, d). Immunofluorescence staining for NANOG and OCT4 showed that the number of NANOG-positive cells was decreased in *Cbx7*-depleted mESCs, while OCT4 remained evenly expressed (Fig. 4f, g). Therefore, CBX7 is indeed required for the proper expression of NANOG and the maintenance of naïve pluripotency. To further confirm that the downregulation of NANOG expression was induced by the loss of CBX7 non-classical function, we induced *Ezh2* deletion in *Ezh2*^f/f^CreERT2 mESCs by 4-OHT. The deletion of *E*zh2 led to the loss of H3K27me3 which is vital for CBX7 classical functions (Additional file 1: Fig. S5d, S6b). Nevertheless, the NANOG expression remained unaffected by *Ezh2* deletion (Additional file 1: Fig. S5e, f), which is consistent with the previous reports showing that PRC2 is dispensable for the maintenance of pluripotency [40–42]. Taken together, the non-classical function of CBX7 in mESCs is biologically significant for the maintenance of pluripotency, which is independent of its classical function.

## Discussion

Although the emerging cases suggest that the non-classical functions of HMRs can be essential to certain cellular processes, there is no framework designed for identifying such functions systematically. In this study, we presented ncHMR detector, the first computational framework to predict the non-classical functions and cofactors of a given HMR systematically, based on ChIP-seq data integration. The framework relies on the significantly enriched co-occurrence of binding events of cofactors and the absence of classical substrates/products of each given HMR, and its cofactor identification has high specificity and robustness. We applied ncHMR detector to ChIP-seq data-rich cell types and predicted 12 non-classical functions of HMRs and their cofactor candidates. Among the top 10 predicted candidates, 2 cases were already partially reported. With the hints of predicted cofactor candidates and public data reanalysis, the understanding of functional mechanisms of both cases was extended. Furthermore, we experimentally validated the predicted non-classical function of CBX7 in mESCs, which is biologically significant for the maintenance of pluripotency, with NANOG as the cofactor. Taken together, the prediction from ncHMR detector not only effectively indicates the existence of non-classical function for a given HMR, but also provides valuable information for its mechanistic investigation. The source code of ncHMR detector and the prediction results are publicly available, which provides a valuable resource for researchers on the non-classical regulatory functions of HMRs.

Despite the aforementioned advantages, ncHMR detector has some technical limitations. First, to effectively identify cofactors, ncHMR detector requires the availability of large amounts of ChIP-seq data for factors within the same cell type, which limited the applicability of the ncHMR detector to a few ChIP-seq data-rich cell types [43, 44]. This limitation could be partially solved by applying chromatin accessibility profiling and motif scanning to predict binding sites of a series of TFs in certain cell types. Second, to effectively avoid the influence of non-specific signal presence or absence in single ChIP-seq data, ncHMR detector was designed to rely on the significantly enriched co-occurrence of binding events of cofactors and the absence of classical substrates/products of each given HMR, which may sacrifice the sensitivity of the framework, especially for cofactors that only bind to a small fraction of the given HMR’s non-classical sites. For example, we previously reported that SETDB1 can modulate PRC2 activity at developmental genes independently of H3K9me3 in mESCs [8]. However, this case cannot be predicted by ncHMR detector, because PRC2 complex members such as EZH2 bind to only 7.1% of SETDB1 non-classical binding sites. We hope our work will overcome this limitation by balancing the specificity and sensitivity of ncHMR detector. Third, ncHMR detector cannot be applied to identify non-classical functions of some HMRs, such as histone acetyltransferases and deacetylases, which have too many known substrates/products, due to the difficulty to define real non-classical binding sites given limited ChIP-data available on the known substrates/products. Fourth, each HMR can have multiple non-classical functions cooperating with distinct cofactors. Our computational framework can report different subsets of non-classical sites for different cofactors, but it does not report whether different cofactors represent distinct non-classical functions. Users could further analyze the lists of non-classical sites for different cofactors to classify the potential multiple non-classical functions of a given HMR.

Although ncHMR detector was designed to identify non-classical functions of HMRs, its application could be extended to other scenarios, in which a TF or HMR has at least two context-dependent functions. One potential scenario is to predict the epigenetic context-dependent TF binding, in view of epigenetic modifications which can explain cell-type-specific binding of many TFs [45]. By treating epigenetic modification differences between two cell types as matrix *X* and the difference in TF binding signals as response *Y* for each given TF, ncHMR detector may be applied to identify key epigenetic modifications that contribute to cell-type-specific binding of some regulatory TFs. Another potential scenario is to predict the cell-type-specific cobinding TF pairs, considering that many TFs cooperate with one another to occupy target genome loci and shape gene expression programs in a cell-type-specific manner [46]. It would be efficient to select cell-type-specific cobinding TF pairs by treating the difference in the co-occurrence of binding events with other TFs between two cell types as matrix *X* and the difference in TF ChIP-seq signals as *Y* for each given TF. Future versions of ncHMR detector could be extended to address a variety of questions related to regulatory complexity.

## Conclusions

Although more and more studies have revealed the biological importance of non-classical functions of HMRs, there are no methods designed for identifying such functions based on ChIP-seq data integration. Here, we developed ncHMR detector, a computational framework for predicting the non-classical functions and cofactors of a given HMR systematically, based on a regression model. We applied ncHMR detector to 6 ChIP-seq data-rich cell types and predicted 12 non-classical functions of HMRs and their cofactor candidates. Moreover, we experimentally validated the predicted non-classical function of CBX7 in mESCs with NANOG as the cofactor. Our study provides a valuable resource for the identification of non-classical functions of HMRs, which will assist researchers to understand the Janus-faced role of HMRs in biological processes well.

## Methods

### ChIP-seq data collection

We collected and filtered ChIP-seq data of HMRs, TFs, and HMs in four ChIP-seq data-rich cell types, including GM12878, K562, hESCs, mESCs, HeLa, and HepG2. We downloaded ChIP-seq peak files, detected by MACS2 [47], of HMRs and TFs, and big wiggle format files (presenting normalized reads density at each genomic loci) of HMs from Cistrome data browser [48]. Only the ChIP-seq data passing at least four out of the first five QCs (i.e., sequence quality, mapping quality, library complexity, ChIP-enrichment, and signal to noise ratio) available in Cistrome data browser were kept. We also filtered out the ChIP-seq data of HMRs with fewer than 1000 peaks. For a factor in matrix *X* or a HM in response *Y*, if it has multiple ChIP-seq data available in a given cell type, we kept only the dataset with the best quality based on QC assessment.

We integrated ChIP-seq data of HMRs, HMs, and TFs passing QC as follows. For each HMR ChIP-seq data, we collected its peak file and the bigwig files of its known HM substrates/products to calculate HM signals surrounding HMR peaks (vector *Y*), whereas ChIP-seq peak files of TFs and other HMRs in the same cell type were integrated to obtain the cobinding occurrence of those factors with the HMR (matrix *X*).

### Workflow of ncHMR detector

The workflow of ncHMR detector consists of the following four steps.

In the first step, the average HM signals across ± 5 kb (for H3K9me3, H3K27me3, and H3K36me3) or ± 1 kb (for other HMs) flanking each ChIP-seq peak center of given HMR were stored in a vector *Y*. We denoted the histone modification signals at *n* ChIP-seq peaks of given HMR as *Y* = (*Y*_1_, …, *Y*_*n*_)^⊤^. The design matrix *X* was generated to represent the cobinding occurrence (0 or 1) with other factors (including TFs and other HMRs) at each ChIP-seq peak of given HMR. The matrix for *p* factors was denoted as $X={\left({X}_1^{\top },\dots, {X}_p^{\top}\right)}^{\top }$, where *X*_*j*_ = (*X*_1*j*_, …, *X*_*nj*_)^⊤^, for *j* = 1, …, *p*, represents the cobinding of factor *j* and the given HMR, with *X*_*ij*_ ∈ {0, 1}, for *i* = 1, …, *n*.

In the second step, ncHMR detector relies on the significantly enriched co-occurrence of binding events of cofactors and the absence of classical substrates/products of each given HMR, and we posited that the response vector *Y* and some columns of *X* are negatively associated in the form of the following linear model (1).

1 $$ {Y}_i={\beta}_0+{\sum}_{j=1}^p{\beta}_j{X}_{ij} $$

We then applied popular feature selection methods, either elastic net^17^ or Lasso^18^, to the linear model (1) to filter out redundant or non-significant factors. The estimation of *β* by elastic net is determined by

2 $$ \hat{\beta}=\underset{\beta }{argmin}\left\{\frac{1}{2}{\sum}_{i=1}^n{\left({Y}_i-{\beta}_0-{\sum}_{j=1}^p{\beta}_j{X}_{ij}\right)}^2+\lambda {\sum}_{j=1}^p\left(\alpha {\beta_j}^2+\left(1-\alpha \right)\left|{\beta}_j\right|\right)\right\} $$

whereas the Lasso estimator is simply a special case of the elastic net estimator by setting *α* = 0. In this paper, we set *α* = 0.5 in the elastic net model. In practice, we used glmnet package^37^ in R to implement both elastic net and Lasso.

In the third step, for each of the remaining factors after feature selection, a univariate linear regression was refitted between the cobinding occurrence of the factor vector *X*_*j*_ and response vector *Y*. Refitting linear regression after model selection by elastic net or Lasso is now common statistical practice with theoretically justified guarantees [49]. If one factor showed strong negative correlation (adjusted *R*^*2*^ > 0.1, where the adjustment was calculated by Wherry’s formula), we permuted the cobinding events between the factor and the given HMR for 1000 times, to test the significance of the calculated correlation coefficient. For each permuted cobinding event, a univariate linear regression was fitted and an adjusted *R*^*2*^ was calculated. The significance of adjusted *R*^*2*^ calculated based on the original cobinding events was defined as the percentage of permutations having adjusted *R*^*2*^ larger than the original un-permuted adjusted *R*^*2*^ (i.e., permutation *p* values). In this study, 0.01 was set as the *p* value threshold to screen factors. If one or more factors showed strong and significant negative correlations, the given HMR was regarded as having a potential non-classical function, and those factors were predicted to be cofactor candidates.

In the fourth step, to report the genomic loci with predicted non-classical functions, the ChIP-seq peaks of the given HMR were classified into classical (the classification with high HM signals) and non-classical (the classification with low HM signals) binding sites using Otsu’s method, an image processing algorithm [23], which calculated the optimum threshold separating the two classifications so that their intra-class variance was minimal. Considering that an HMR may cooperate with multiple cofactors independently, the framework reported subsets of the non-classical sites overlapping with the binding sites of each cofactor candidate.

### Performance evaluation based on simulation data

To evaluate the prediction performance of ncHMR detector, we designed two setups for specificity and robustness evaluation based on simulation data. The first setup is designed for specificity evaluation of cofactor identification. Based on EZH2 and H3K27me3 ChIP-seq data in mESC (GSM1199182, GSM1199183, GSM1399500, GSM1399503), we simulated the cobinding events with EZH2 for four groups of other factors, and different groups displayed distinct correlations (strong negative, weak negative, weak positive, and strong positive, separately) between cobinding occurrence (0 or 1) with EZH2 and response H3K27me3 signals. The strong negative group included 15 factors with a negative correlation coefficient with H3K27me3 and adjusted *R*^*2*^ > 0.1, and the weak negative group included 35 factors with a negative correlation coefficient and adjusted *R*^*2*^ < 0.1. Similarly, the strong positive group included 15 factors with positive correlation coefficient with H3K27me3 and adjusted *R*^*2*^ > 0.1, and the weak positive group included 35 factors with positive correlation coefficient and adjusted *R*^*2*^ < 0.1. We generated 5 simulation datasets in this study. Based on each simulation dataset, the specificities of ncHMR detector and approaches using other feature selection methods, including forward selection [30] and knockoff [31], were calculated by treating the factors in the strong negative group as true positive cofactors and other factors as true negative. In addition, the specificity was also measured by the frequency of cobinding events between predicted cofactors and EZH2 at the non-classical sites of EZH2.

The second setup is designed to evaluate the robustness of cofactor identification. Three types of noises were added to the evaluation dataset. First, to simulate the experimental variation on HM ChIP-seq data, we added a Gaussian noise on H3K27me3 signal at EZH2 ChIP-seq peaks. In each simulation, the mean and standard deviation of the noise distribution were set to be equal to a given fraction of the average histone modification signal across the genome. The fraction was set from 0.1 to 1, with 0.1 as the interval. Second, to simulate the experimental variation on HMR ChIP-seq data, we randomly omitted a percentage of non-classical sites of EZH2 (from 10 to 80%, with 10% as the interval). Third, to throw away the experimental variation on other factors’ ChIP-seq data, we randomly altered a percentage of cobinding events (alteration from 1 to 0, or from 0 to 1) with EZH2 for other factors (from 2 to 20%, with 2% as the interval). For the above three types of noise, we generated 10 simulated datasets for each case. We used specificity and F-beta score (*β* = 0.75) to evaluate the robustness of cofactor identification in each simulation condition, by treating the factors in the strong negative group as true positive cofactors and other factors as true negative.

### Comparison with modified existing methods

The following existing methods can be modified to predict cofactors of non-classical functions of HMRs: (1) MultiGPS, (2) edgeR, (3) DBChIP, and (4) factor co-association analysis. MultiGPS [19], edgeR [20], and DBChIP [21] can be applied to detect differential binding events across multiple conditions, which were related, but distinct to identify its context-dependent binding sites in a given condition. In the method comparison part, we modified the aims of those methods by identifying differential enriched regions between a given HMR and its HM substrates/products ChIP-seq data, and defined the specifically enriched regions in the HMR ChIP-seq data (fold change > 20 and FDR < 0.01) as its non-classical sites. To identify the cofactors of non-classical function, we modified those methods by applying Jaccard index to compute the overlapping frequency between potential cofactors and identified non-classical sites of HMR, and those with Jaccard index > 0.35 were defined as cofactors of the non-classical functions. Gerstein et al. developed context-specific TF co-association analysis method (https://code.google.com/archive/p/tf-coassociation/source/default/source) [22], and this method can be used to identify partner factors with co-occurrence of binding events at specific subsets of a given HMR’s binding sites. As it cannot distinguish cofactors of classical and non-classical functions of a given HMR, we added a column containing the presence/absence status of its HM substrates/products to the input cobinding matrix. In that column, HMR binding sites with HM peaks were assigned to 0 and other HMR binding sites were assigned to 1. Factors which showed high-confidence co-associations (*CS* ≥ 5) with the feature of HM absence were identified as cofactors of non-classical functions of the given HMR.

In the method comparison between ncHMR detector and four modified existing methods, we applied the same simulation datasets used in performance evaluation of ncHMR detector. As the simulation of the experimental variation on HM ChIP-seq data is not applicable in some modified existing methods (MultiGPS + Jaccard index, edgeR + Jaccard index, and DBChIP + Jaccard index), only simulations of experimental variation on HMR ChIP-seq data and other factors’ ChIP-seq data were used in method comparison.

In the comparison of ncHMR detector and modified factor co-association analysis, we also generated simulated cobinding matrix with quantitative values (ranging from 0 to 1) as follows. Based on the approach used to generate quantitative cobinding matrix in Gerstein et al., for all 0 (no cobinding) in binary cobinding matrix, we kept it as 0 in the quantitative cobinding matrix, and for all 1 (cobinding) in binary cobinding matrix, we simulated an intensity rank for the overlapped binding sites and computed the normalized peak intensities (ranging from 0 to 1) used in the quantitative cobinding matrix. For the additional column representing the presence/absence status of HM in the modified factor co-association analysis, we ranked HM signals at all HMR binding sites and reversed the rank to compute the normalized peak intensities.

### Statistical analysis

In the factor selection step, we used standard elastic net to predict histone modification signals on HMR peaks with the cobinding events of different factors. By default, we set *α =* 0.5 to control the relative weighting of *Lasso* and *Ridge*. Besides, we used the value of *lambda.1se* as the selected value for *λ* in order to provide a simpler model with comparable error to the best model. The cofactor candidates with non-zero coefficient are selected as significant factors in the feature selection step.

### Software and webserver implementation

The ncHMR detector software was implemented using Python and R, under GNU General Public License v2.0. It is available at https://github.com/TongjiZhanglab/ncHMR_detector. We used glmnet package in R to implement the regularized regression (elastic net and Lasso) in the feature selection step. Users can change the default values of *α* and *λ* by setting the parameters *--Alpha* and --*LambdaChoice*. In the non-classical function qualification step, we used the built-in lm function in R to implement the univariate linear regression. By default, we set a stringent *R*^*2*^ cutoff (0.1) with an empirical *p* value cutoff (0.001) to report the predicted candidates. Users can also change *R*^*2*^ cutoffs by setting the parameter --R-squared. The higher stringent *R*^*2*^ cutoffs lead to less candidates. The software reports the candidates ranking from highest *R*^*2*^ to the lowest. Besides, the ncHMR detector software provides another running mode (using quantitative values in matrix *X*) by setting the *parameter --mode* as *signal*. In that running mode, the values in matrix *X* are the average signals of TF ChIP-seq data at the given HMR’s peaks. Bigwig files of TFs are required under that running mode.

The ncHMR detector webserver was implemented using HTML, JavaScript, and PHP, and it is freely available at http://compbio-zhanglab.org/ncHMR_detector/index.php. The usage instructions and example files are provided at the website.

### Hi-C data processing

Processed Hi-C contact matrix in hESC was sourced from [50] and downloaded from 4D nucleosome data portal. Raw Hi-C data in mESC were obtained from Du et al. [51]. Raw reads were aligned, processed by using HiC-Pro [52]. Pairs of aligned reads were then assigned to MboI restriction fragments. Read pairs from uncut DNA, self-circle ligation, and PCR artifacts were filtered out, and the valid read pairs involving two different restriction fragments were used. Valid read pairs in hESC and mESC were both dumped with KR normalization at 5000-bp resolution to examine local interactions by using Juicer [53].

### Cell culture and stable cell generation

The mESCs were cultured on 0.1% gelatin-coated plates in 2i medium (F12/Neuralbasal medium 1:1, non-essential amino acids, l-glutamine, β-mercaptoethanol, penicillin/streptomycin, sodium pyruvate, N2/B27 and leukemia inhibitory factor (LIF), GSK3β and MEK1 inhibitors), or SL medium (GMEM, non-essential amino acids, l-glutamine, β-mercaptoethanol, penicillin/streptomycin, sodium pyruvate, 15% fetal bovine serum, and LIF). All cell cultures were maintained at 37 °C with 5% CO_2_.

To knockdown *Cbx7*, specific oligonucleotides (GTGAAGTTACCGTGACTGA) were designed and cloned into pLKO.1 TRC cloning vector according to the protocol recommended by Addgene. The shRNA expressing constructs were co-transfected with pAX8 (packaging) and pCMV-VSVG (enveloping) into 293FT cells. After 48 h, virus supernatants were harvested and mESCs were infected along with polybrene (8 μg/ml). Positive cells were selected with 2 μg/ml puromycin 48 h post-infection. To knockout *Cbx7*, specific oligonucleotides (GCATGCTGTACAGCCGCTGCA) were designed and cloned into px459 vector according to the protocol recommended by Addgene. Then the sgRNA-expressing constructs were transfected into mESCs and selected with 2 μg/ml puromycin for 48 h. Single-cell clones were isolated and validated by genome DNA PCR.

### Real-time qPCR

For gene expression analysis, total RNA was extracted by TRIZOL and reverse transcribed by the Reverse Transcription Reagents Kit (Thermo Fisher). All qPCR analyses were performed using the LightCycler® 480 SYBR Green I Master (Roche) with the ABI 7500 fast PCR System (Applied Biosystems). All data were normalized to Rpo. The primer sequences are listed in Additional file 3: Table S2.

### ChIP-qPCR analysis

The ChIP assay was performed as described previously [54]. Briefly, 5 × 10^5^–1 × 10^7^ cells were fixed in 1% formaldehyde (i.e., 15 ml for a 15-cm dish) at room temperature for 10 min. Then, fixation was stopped by the addition of glycine to a final concentration of 0.125 M and incubation for 5 min. The plates were rinsed twice with 1 × PBS at room temperature; then PBS was aspirated completely from the plate and the cells harvested in SDS buffer (100 mM NaCl, 50 mM Tris-HCl, pH 8.1, 5 mM EDTA, 0.02% NaN_3_, 1% SDS) containing protease inhibitors. Cells were pelleted by spinning in a tabletop centrifuge for 5 min at 1800 rpm, then resuspended in ice-cold IP buffer for sonication (IP buffer = 1 volume SDS buffer:0.5 volume Triton dilution buffer (100 mM Tris-HCl, pH 8.6, 100 mM NaCl, 5 mM EDTA, pH 8.0, 0.02% NaN_3_, 5.0% Triton X-100)). Then the samples were sonicated using a Bioruptor for 10 min (30 s on/off per cycle). Sonicated chromatin was pelleted by centrifugation at 20000×*g* for 5 min. Then, 3 μg of antibody (CBX7 ab21873, Abcam) was added to the supernatant (500 μg chromatin for each IP) and rotated overnight at 4 °C. Protein A+G beads were added the next morning for 3 h. Beads were washed once with wash buffer 1 (1% Triton X-100, 0.1% SDS, 150 mM NaCl, 2 mM EDTA, 20 mM Tris-HCl, pH = 8.0) and once with wash buffer 2 (1% Triton X-100, 0.1% SDS, 500 mM NaCl, 2 mM EDTA, pH 8.0, 20 mM Tris-HCl, pH 8.0), then reverse crosslinked at 65 °C overnight. DNA fragments were column-purified (QIAGEN, Cat.NO. 28106) for qPCR analysis. The primer sequences are listed in Additional file 3: Table S2.

### Immunofluorescent staining

The mESCs were fixed in cold methanol for 3 min, washed twice with PBS, and then blocked with 0.8% BSA for 10 min. Antibodies (OCT4: ab184665 Abcam; NANOG: A300-397A Bethyl) were incubated at 37 °C for 1 h, and the secondary antibodies were incubated at 37 °C for another hour. Images were acquired using a laser scanning confocal microscope (ZEISS, LSM800).

## Supplementary information

**Additional file 1.** Supplementary figures (Fig. S1-S6).

**Additional file 2: Table S1.** Predicted non-classical functions and cofactor candidates of HMRs. Each row represents a cofactor candidate ranked by adjusted *R*^*2*^.

**Additional file 3: Table S2.** Primer sequences for qPCR and ChIP-qPCR.

**Additional file 4.** Review history.

## Data Availability

The ncHMR_detector software is an open source software under GNU General Public License v2.0, and it is available at https://github.com/TongjiZhanglab/ncHMR_detector [55] and 10.5281/zenodo.3629352 [56]. Usage instructions and example files are provided in the README section at the webpage. The ncHMR detector webserver is available at http://compbio-zhanglab.org/ncHMR_detector/index.php. Usage instructions and example files are also available at the webserver. The embryonic stem cells used in this article are primary cells derived from ICM of mouse E3.5 embryos. Public datasets for profiling non-classical function of HMRs and simulation analysis can be found at the Gene Expression Omnibus (GEO) under accession numbers GSE11431, GSE22557, GSE27841, GSE34518, GSE49431, GSE55697, GSE58023, GSE64008, GSE67867, GSE73432, GSE90893, and GSE90895 [32, 57–66].

## Abbreviations

HMR: Histone modification regulator
QC: Quality control
ncHMR detector: Non-classical functions of histone modification regulator detector
mESC: Mouse embryonic stem cell
hESC: Human embryonic stem cell
HM: Histone modification
TF: Transcription factor
TSS: Transcription start site
