# Supplementary material for: ncHMR detector: a computational framework to systematically reveal non-classical functions of histone modification regulators
Source: Genome Biol. 2020 Feb 24;21:48. doi: 10.1186/s13059-020-01953-0 (PMC7038559; doi:10.1186/s13059-020-01953-0)
Supplement: Supplementary file 1 — Additional file 1. Supplementary figures (Fig. S1-S6). [file 13059_2020_1953_MOESM1_ESM.pdf]

a

Collection of known non-classical functions

HMR	Histone Modification	Cofactor	ChIP-seq	Reference
RNF2	H2Aub1	KDM1A,KDM2B	Not Available	Sanchez et al, 2007
CBX7	H3K27me3	HDAC2	Not Available	Federico et al, 2009
EZH2	H3K27me3	AKT1	Not Available	Gonzalez et al, 2011
KDM4B	H3K9me3,H3K36me3	KMT2B,RBBP5, ASH2L,WDR5	Not Available	Shi et al, 2011
EZH1	H3K27me3	-	GSE25549	Mousavi et al, 2011
EZH2	H3K27me3	AR	GSE39461	Xu et al, 2012
EZH2	H3K27me3	-	GSE49435	Kaneko et al, 2013
KDM4B	H3K9me3,H3K36me3	KDM4C	GSE43231, GSE75426, GSE51006	Das et al, 2013
KDM4C	H3K9me2,H3K9me3, H3K36me2,H3K36me3	EZH2	GSE43231, GSE75426, GSE51006	Das et al, 2013
EZH1	H3K27me3	-	GSE59090	Xu et al, 2015
SETDB1	H3K9me3	EZH2	GSE17642,GSE75426, GSE62666	Fei et al, 2015
CBX7	H3K27me3	EP300	Not Available	Kim et al, 2015
EZH2	H3K27me3	E2F1	GSE39461,GSE67809	Xu et al, 2016
EZH2	H3K27me3	JAK3	Not Available	Nawaz et al, 2016
RNF2	H2Aub1	MED12	GSE42466, GSE34518, GSE22557	Papadopoulou et al, 2016
CBX8	H3K27me3	WDR5	Not Available	Chung et al, 2016
EZH2	H3K27me3	TRIM28	GSE29611,GSE76271, GSE38788	Li et al, 2017
KDM2	H3K36me2	-	GSE41267,GSE41589	Turberfield et al, 2019

b

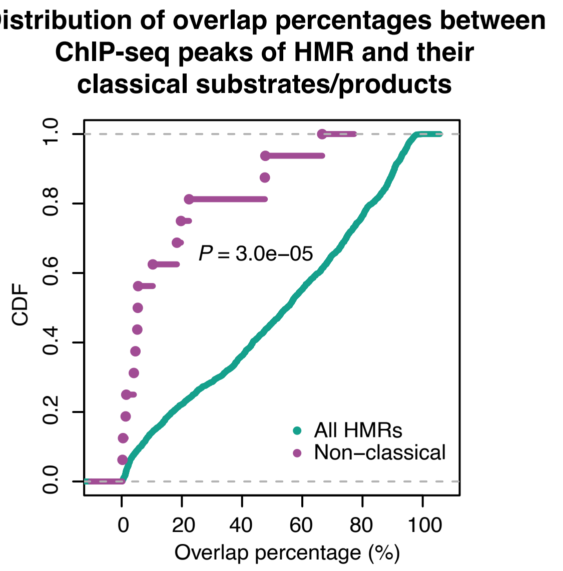

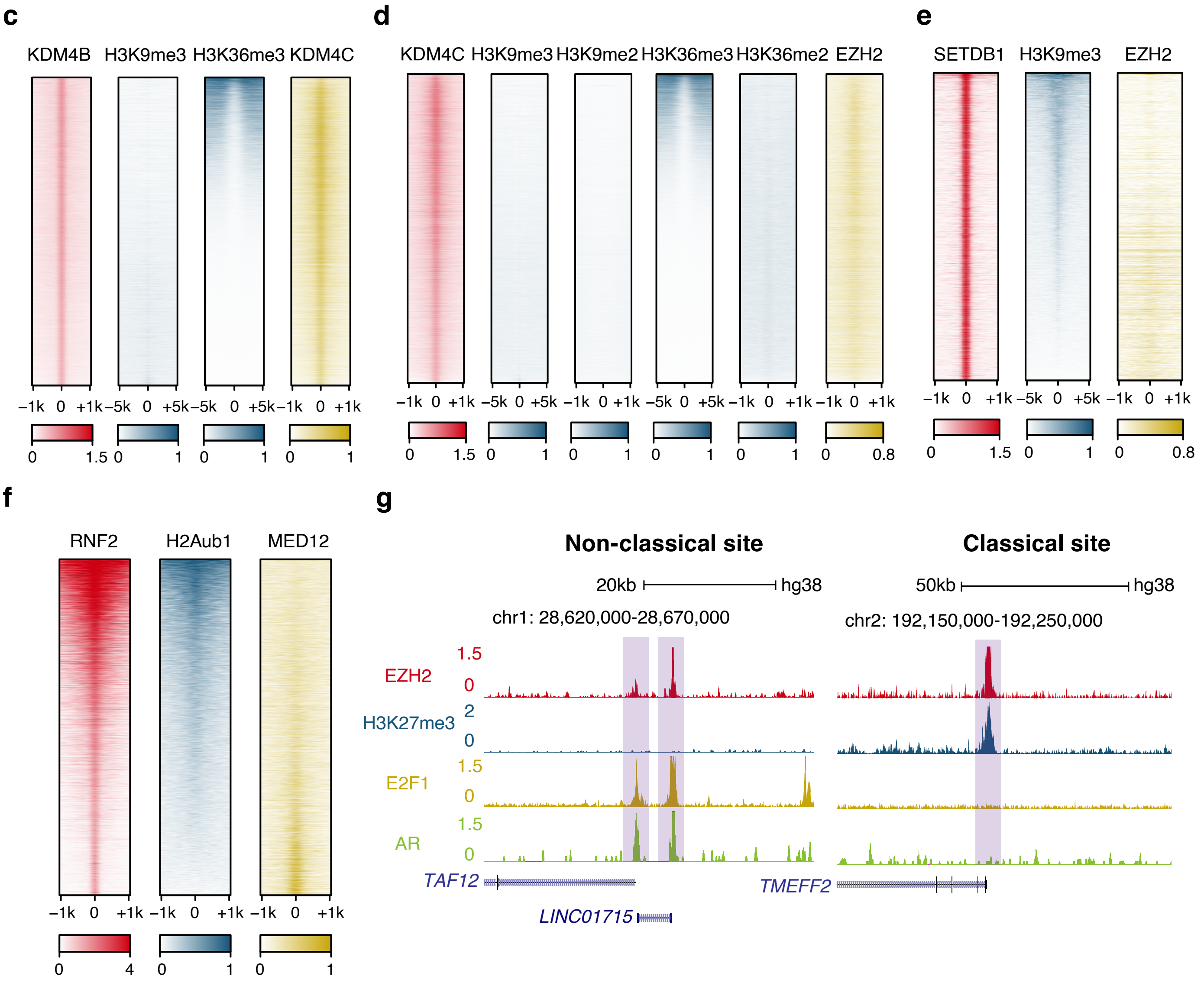

Fig. S1 (related to Fig. 1). (a) Collection of previously reported non-classical functions, including 11 cases discovered by high-throughput ChIP-seq data analysis and 7 cases discovered by low-throughput technologies. (b) Line chart shows the cumulative distribution function (CDF) of overlap percentages between ChIP-seq peaks of HMRs and their classical substrates/products. The green line represents overlap percentages between all HMRs and their classical histone modification substrates/products pairs on CR Cistrome [1]. And the purple line represents overlap percentages between HMRs with reported non-classical functions and their classical substrates/products. The Kolmogorov-Smirnov test was performed to identify statistical significance. (c-f) Heatmap showing HMRs (KDM4B, KDM4C, SETDB1, RNF2), their corresponding classical histone modifications and cofactors enrichment at HMR ChIP-seq peak centers. Rows represent HMR binding sites and are ranked by normalized histone modification signals. The colors indicate the normalized ChIP-seq enrichment level and the values are scaled by row. (g) The UCSC genome browser view of the representative non-classical site (left) and classical site (right) of EZH2, which are shaded in purple. Signals represent ChIP-seq RPM. All ChIP-seq data used for re-analyzing reported non-classical functions were annotated in (a).

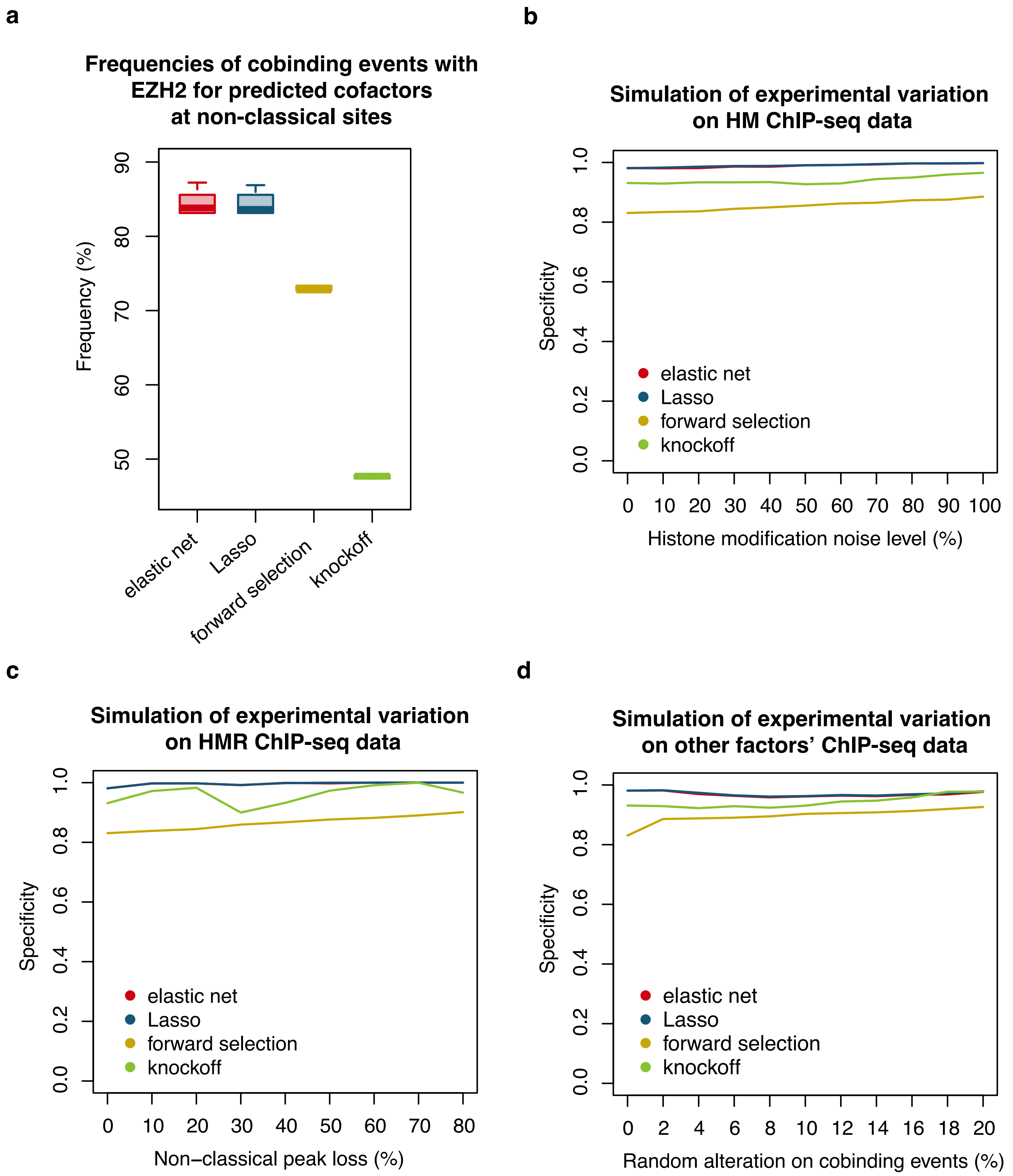

Fig. S2 (related to Fig. 2). (a) Box plot showing the frequencies of cobinding events between predicted cofactors and EZH2 at the non-classical sites of EZH2. The red, blue, yellow and green box represent cofactors identified by elastic net, Lasso, forward selection and knockoff. (b-d) Line charts showing the robustness of cofactor identification on evaluation data with 3 types of noise that simulate 3 kinds of experimental variation on HM (b), HMR (c) and other factors' (d) ChIP-seq data. The specificity was used to evaluate the robustness by treating the factors in the strong negative group as true positive cofactors and other factors as true negative. The red, blue, yellow and green lines represent cofactors identified by elastic net, Lasso, forward selection and knockoff, respectively.

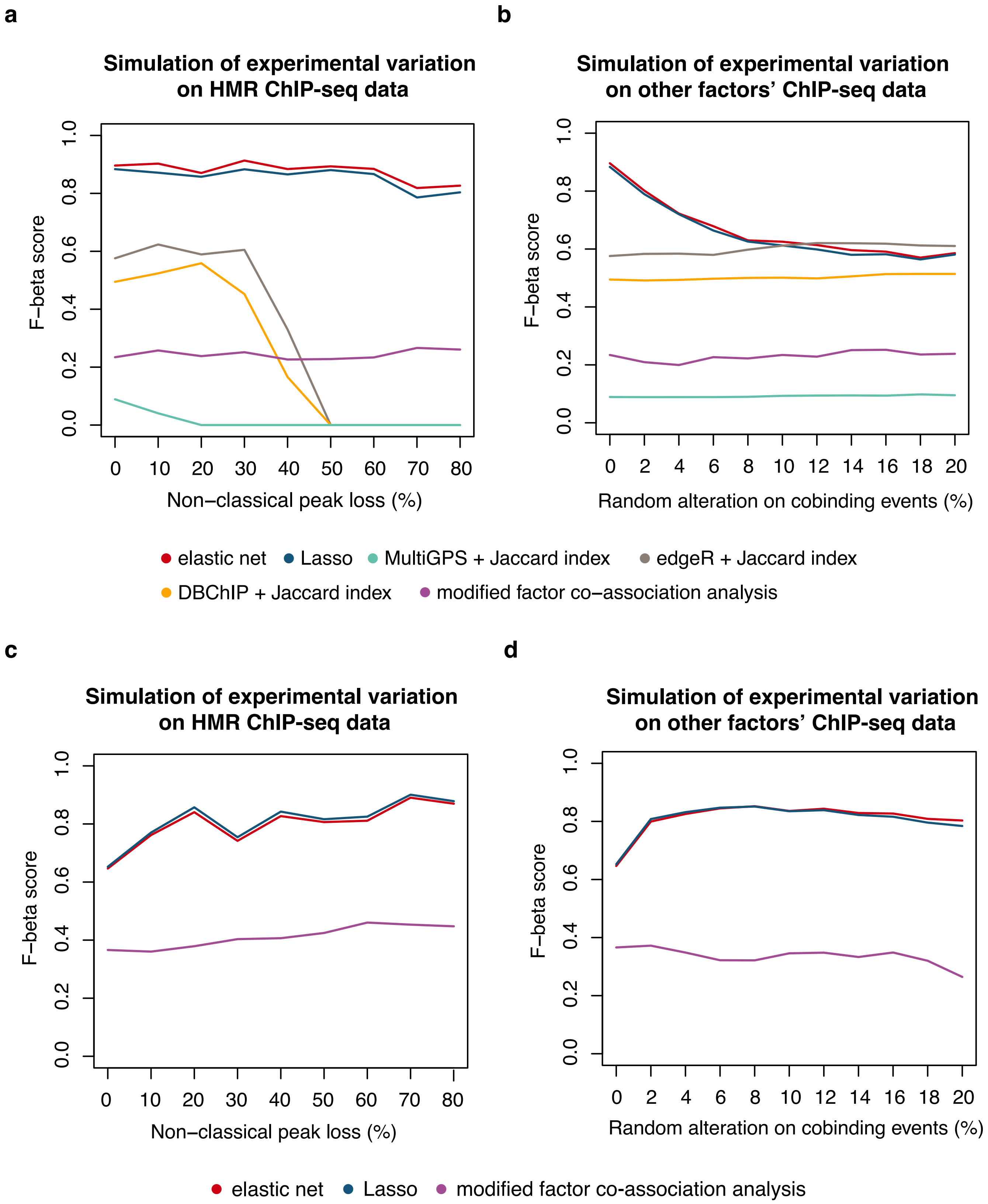

Fig. S3 (related to Fig. 2). (a, b) Line charts showing the robustness of cofactor identification on evaluation data using binary cobinding matrix with 2 types of noise that simulate 2 kinds of experimental variation on HMR (a) and other factors' (b) ChIP-seq data. (c, d) Line charts showing the robustness of cofactor identification on evaluation data using quantitative cobinding matrix with 2 types of noise that simulate 2 kinds of experimental variation on HMR (c) and other factors' (d) ChIP-seq data. The F-beta score ($\beta = 0.75$) was used to evaluate the robustness by treating the factors in the strong negative group as true positive cofactors and other factors as true negative. The red, blue, light green, brown, orange and purple lines represent cofactors identified by elastic net, Lasso, MultiGPS + Jaccard index, edgeR + Jaccard index, DBChIP + Jaccard index and modified factor co-association analysis.

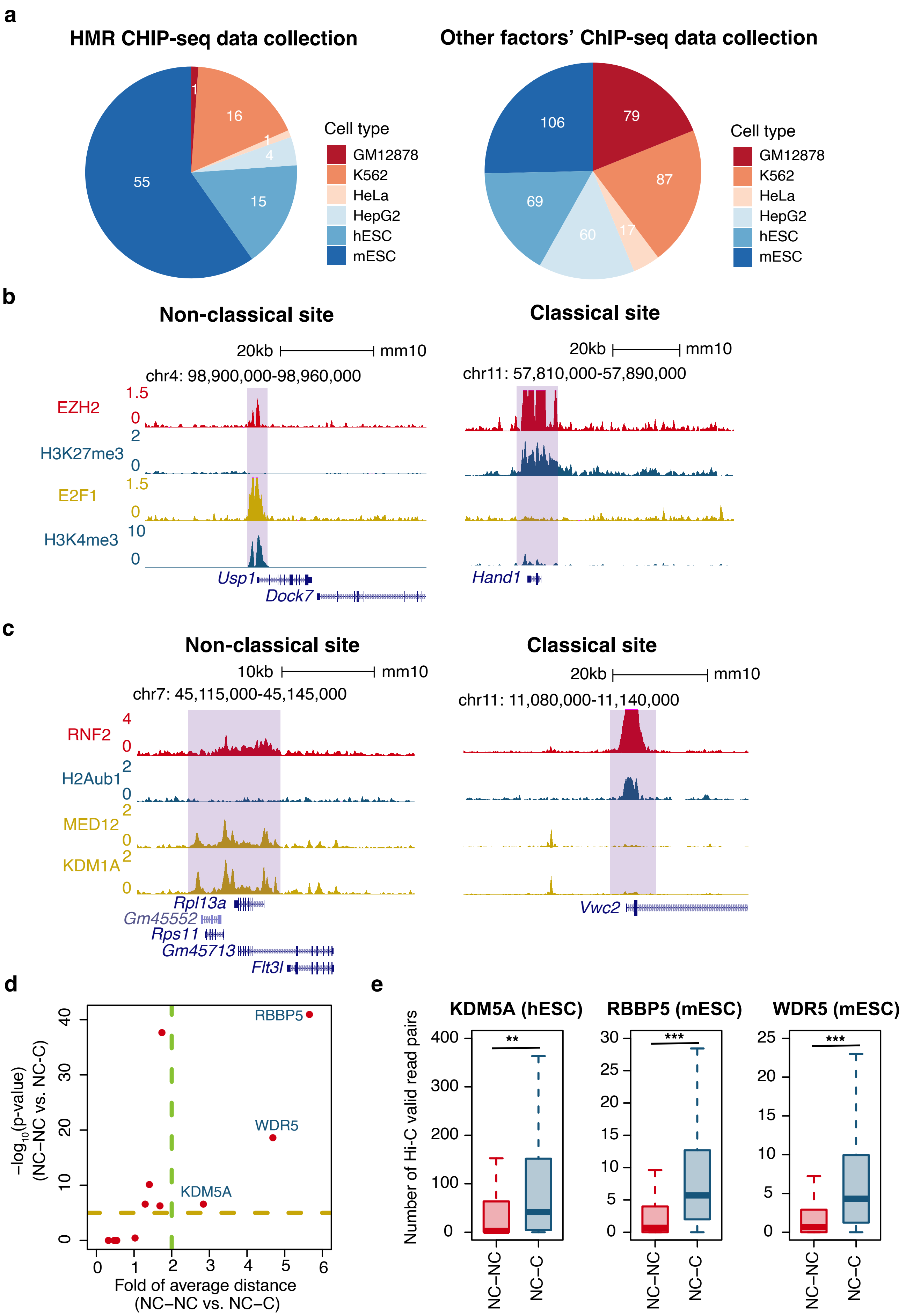

Fig. S4 (related to Fig. 3). (a) The pie chart shows the percentage of collected ChIP-seq data of HMR and other factors in cobinding matrix *X* in 6 data-rich cell types, GM12878, K562, hESCs, mESCs, HeLa and HepG2. (b) The UCSC genome browser view of the representative non-classical site (left) and classical site (right) of EZH2, which are shaded in purple. Signals represent ChIP-seq RPM. (c) The UCSC genome browser view of the representative non-classical site (left) and classical site (right) of RNF2, which are shaded in purple. Signals represent ChIP-seq RPM. (d) The comparison between the average distance of non-classical sites (NC) to the nearest classical sites (C) and to the nearest other non-classical sites. X axis represents fold of the average distance (NC-NC: non-classical sites to the nearest other non-classical sites, *vs.* NC-C: non-classical sites to the nearest classical sites). Y axis represents the $-\log_{10}$ transformed *p*-value (calculated by using unpaired t test). Green dashed line represents fold threshold 2, yellow dashed line represents *p*-value threshold 10^{-5} and HMRs with fold > 2 and *p*-value < 10^{-5} were labelled in blue text. (e) Box plot comparing the numbers of Hi-C valid read pairs between non-classical sites and other non-classical sites (NC-NC), and between non-classical sites and classical sites (NC-C). Unpaired t test was used to identify statistical significance for comparison (* represents *p*-value < 0.05, ** represents *p*-value < 0.01, *** represents *p*-value < 0.001 and n.s represents non-significant).

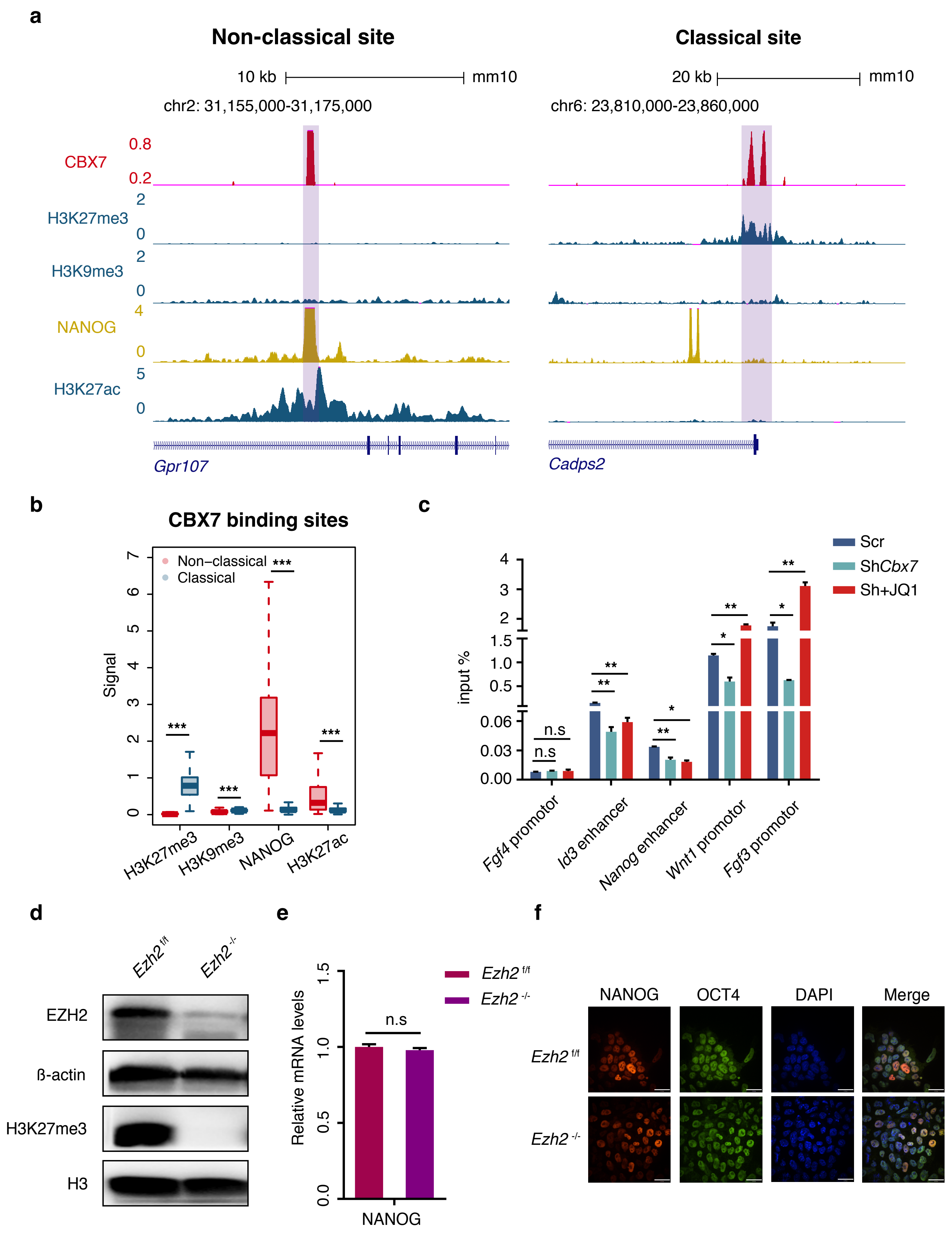

Fig. S5 (related to Fig. 4). (a) The UCSC genome browser view of the representative non-classical site (left) and classical site (right) of CBX7, which are shaded in purple. Signals represent ChIP-seq RPM. (b) Box plot comparing H3K27me3, H3K9me3, NANOG and H3K27ac enrichment levels at non-classical sites and classical sites of CBX7. The Wilcox test was used to identify statistical significance for comparison (* represents *p*-value < 0.05, ** represents *p*-value < 0.01, *** represents *p*-value < 0.001 and n.s represents non-significant). (c) ChIP-qPCR analysis for CBX7 at non-classical sites (*Id3* enhancer, *Nanog* enhancer) and classical sites (*Wnt1* promoter and *Fgf3* promoter). A non-bindsing site (*Fgf4* promoter) was used as a control. Error bars represent the standard deviation for triplicate experiments and unpaired t test with Welch's correction was used to calculate statistical significance for comparison (* represents *p*-value < 0.05, ** represents *p*-value < 0.01, *** represents *p*-value < 0.001 and n.s represents non-significant). (d) Western blot validates the knockout of *Ezh2* and shows the decrease of H3K27me3 in *Ezh2*$^{-/-}$ mESCs. Blots were cut before antibody application. Gel images of Western blot are shown in Additional file 1: Fig. S6b. (e) Real-time qPCR analysis for the expression of NANOG in *Ezh2*$^{f/f}$ and *Ezh2*$^{-/-}$ mESCs. Error bars represent the standard deviation for triplicate experiments and unpaired t test with Welch's correction was used to calculate statistical significance for comparison. (f) Immunofluorescence staining shows no obvious decrease of NANOG or OCT4 in *Ezh2*$^{-/-}$ mESCs compared to *Ezh2*$^{f/f}$ mESCs.

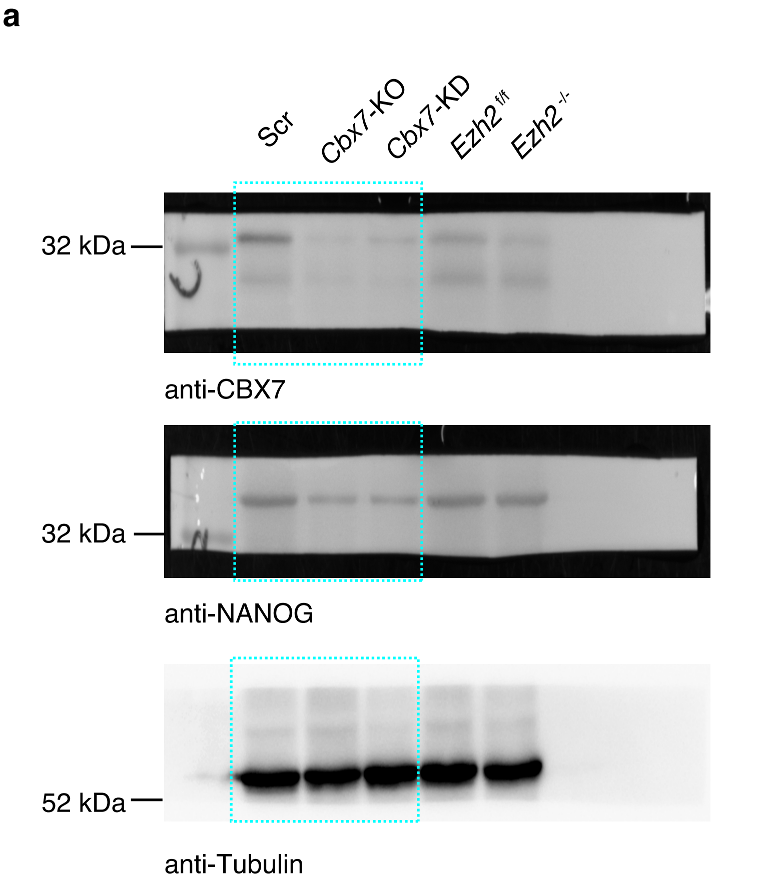

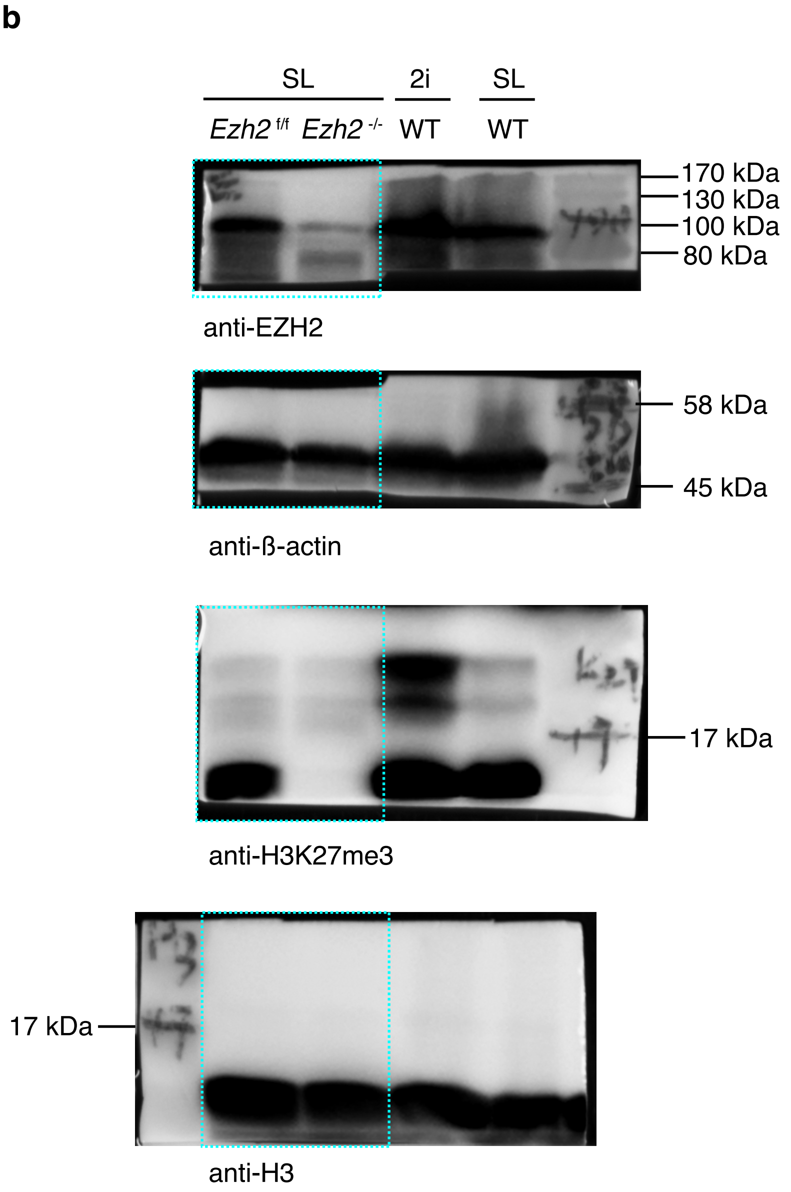

Fig. S6 Gel images for Western blot analyses in Fig. 4d and Additional file 1: Fig. S5d. (a) Images for Western blot analysis of CBX7 and NANOG level in wild type, *Cbx7*-knockout and *Cbx7*-knockdown mESCs. (b) Images for Western blot analysis of EZH2 and H3K27me3 level in *Ezh2* $^{f/f}$ and *Ezh2* $^{-/-}$ mESCs. Blots were cut before antibody application. All blots used in this study are shown in blue dashded box.

Reference

1. Wang Q, Huang J, Sun H, Liu J, Wang J, Wang Q, et al. CR Cistrome: a ChIP-Seq database for chromatin regulators and histone modification linkages in human and mouse. Nucleic Acids Res. 2014;42(Database issue):D450-8.
