# Supplementary material for: ncHMR detector: a computational framework to systematically reveal non-classical functions of histone modification regulators
Source: Genome Biol. 2020 Feb 24;21:48. doi: 10.1186/s13059-020-01953-0 (PMC7038559; doi:10.1186/s13059-020-01953-0)
Supplement: Supplementary file 4 — Additional file 4. Review history. [file 13059_2020_1953_MOESM4_ESM.docx]

Review History

**First round of review**

**Reviewer 1**

**Comments to author:**

Major Issues:

There are several similar tools published. please see below.

i.     DeepHistone
ii.     DeepChrome
iii.     Epigram
iv.     ChIPSeqR
v.     EpiRegNet
vi.     Epidauraus
vii.     HMCan
All of which predict histone modifications given a DNA sequence or gene expression data as input. I would like the authors to mention how their ncHMR detector outstands these available conventional tools. I recommend the authors to add a section in the introduction section supporting their statement.
b.     The Abstract should be more descriptive, and the English language should be better phrased especially in regard to the grammar and sentence construction.
c.     The link provided is a GitHub repository. Would it be possible for the authors to develop a web resource that allows users analyze their data sets. This would be key for biological researchers who do not have bioinformatics experience.
d.     When incorporating ChIP-Seq data sets. My major concern is why did the authors not consider querying the ENCODE database? I am certain the authors would have retrieved a larger data set in comparison to what they obtained primarily.
e.     The authors mention that the ChIP-Seq data was "integrated" (page 14). The authors must provide more description as to how they performed the integration.
f.     The authors use a lot of machine learning or deep neural network algorithms in their ncHMR detector. It is extremely important for the authors to explain they performed this analysis on the whole downloaded dataset or had they divided the data into training, validation and test sets in order to confer their model and check the accuracy.
g.     Page 20. The following headings are missed in the methods sections. The headings include; statistical analysis (what packages they utilized), developing the ncHMR detector or data availability. It would also be good to have a web source heading that describes how the public resource was developed.  I recommend the authors to include these sections.

Minor comments:
a.     Page 5 - ABL has not been expanded when used in the first instance.
b.     Page 6 - The link has already been mentioned in the introduction section. This should be removed.
c.     Page 6, 8, 9, 12 and 14 - The authors use terms such as "important factors", "other methods" and "dozens". These terms should not be used in research articles. It would be good to see the authors explain the results section in detail (for. e.g. what factors or methods were used or compared, how many "numbers" of non-classical functions were obtained)
d.     Page 7 - Specificity using greedy forward selection and knock off values should be either represented separately as supplementary figures or a reference could be provided for the analysis results.
e.     Page 10 - The authors use a term "CHROMO". It is not clear as to what the authors are referring to.

**Reviewer 2**

**Comments to author:**

This manuscript describes ncHMR detector that is designed to identify non-classical functions of HMRs. ncHMR detector relies on the significantly enriched co-occurrence of binding events of cofactors and the absence of classical substrates/products of the given HMR, and requires the availability of a large amount of ChIP-seq data for other factors in the same cell type. The proposed method was applied to the ChIP-seq data for GM12878, K562, hESCs and mESCs and in total 14 non-classical functions of HMRs were predicted. One of them, the non-classical function of CBX7 in mESCs was biologically validated. The manuscript could be of use to the field. My concerns are mainly about technical points elaborated on below.

1)     Six of the top-10 HMRs (Fig. 3A) have broad marks (H3K27me3 and H3K9me3) as classical substrates/products. It may be inappropriate for such broad marks to focus on the small width (±1 kb flanking each ChIP-seq peak center of given HMR) because broad marks have much weaker S/N ratio than sharp peaks (e.g., H3K4me1) that makes difficult to distinguish whether each region is enriched or not. Does the proposed program assume the sharp and strong peaks of these marks? The discussion is necessary.
2)     Human and mouse ES cells are exceptional cases in which H3K27me3 is highly enriched around "poised promoter" TSSs. Because eight of top-10 HMRs (Fig. 3A) were from ES cells, the more results from other cell lines (e.g, GM12878, HeLa or HepG2) are required.
3)     As related works for more general purposes, Gerstein et al. analyzed many TF ChIP-seq data and defined primary and local partners for each TF to identify context-specific function of TFs (Gerstein et al, "Architecture of the human regulatory network derived from ENCODE data", Nature 2012). There are also many tools for "joint-peak analysis" (e.g. MultiGPS http://mahonylab.org/software/multigps/). While these are not designed for HMRs, they can be used. Please clarify the novelty of the proposed method against these existing studies.
4)     While the signals of HM (vector Y in Fig. 1d) are calculated quantitatively, TF peaks (matrix X in Fig. 1d) are evaluated binary (appear or not). Why does not the proposed method treat the signal of TF peaks quantitatively? That makes possible to evaluate not only the peak existence but also the quantitative change of shared peaks.
5)     The proposed method simply uses peaks identified by MACS2 but it depends on the number of mapped reads. Therefore the sensitivity and specificity of the proposed method is dependent on the read number of the TF ChIP-seq data used. Taken together with my comment 3, quantitative value is desirable for TF peaks.
6)     Each HMR can have two or more non-classical functions other than the classic function, while the proposed method may not be able to distinguish them. Please add the discussion for this point.
7)     In Fig 4a, some of CBX7 peaks colocalized with H3K27me3, H3K9me3 and NANOG. Does this result suggest NANOG also functions in the classical CBX7 peaks?
8)     Though the evaluation of the non-classical function of CBX7 confirmed the effectiveness of the proposed method, the other predicted functions (Fig. 3a) also need to be discussed referring the existing biological studies. For example, CTCF was identified as a cofactor of KMT2B, but CTCF itself has many cofactors such as cohesin (Wendt. Nature 2008). Why were not the cofactors of CTCF identified in this analysis whereas the cohesin ChIP-seq data for mESC is available?
9)     In some cases, non-classical sites and classical sites may be functionally correlated (e.g. enhancer and promoter sites for same genes). Do the authors have comments about the way to distinguish whether identified non-classical function is correlated with the classical one? One possibility may be the averaged distance between the sites.
10)     In addition to Fig. S4e, the RT-qPCR result for the expression of NANOG in Ezh2 +/+ and Ezh2 -/- is necessary (same as Fig. 4C).
11)     The main manuscript is a bit redundant. Especially, several sentences in Conclusion were very similar to text in Discussion.

Minor:
1)     I strongly recommend updating the proposed method from Pyhon2 to Python3 because Python2 will retire in the next year.
2)     Please add the time of experiments and the p-value to show the statistical significance for Fig. 4c and Fig. S4c.

**Reviewer 3**

This is an interesting study with a novel tool, ncHMR detector, which is the first computational framework to predict the non-classical functions and cofactors of a given HMR systematically, based on ChIP-seq data integration. It is an important question to identify the non-classical functions of HMRs due to its essential involvement in a variety of cellular processes. However, there is no framework designed for identifying such functions systematically except few cases reported in the literature. Generally speaking, this is a nice study except few questions listed below:
Major:
1.     To some extent, it is hard to evaluate the prediction accuracy of the tool though the author has done few confirmations. What is the FDR for ncHMR detector? Will it be affected by your choice of R cutoff?
2.     What is the rationale to choose R<0.1?
3.      In the second step, what cutoff was used to select significant factors with feature selection by elastic net or Lasso?
Minor: In fig. 4, Cbx7-knockout (KO) has less effect on Nanog expression compared with Cbx7-knockdown, confirmed by both Western blot (fig. 4d) and immune-staining (fig. 4f). Any explanation? Cbx7-KO should have more profound effect on Nanog expression compared with partial knockdown of Cbx7.

**Authors Response**

**Point-by-point responses to the reviewers’ comments:**

Reviewer #1:

Major Issues: a. There are several similar tools published. please see below. i. DeepHistone ii. DeepChrome iii. Epigram iv. ChIPSeqR v. EpiRegNet vi. Epidauraus vii. HMCan

All of which predict histone modifications given a DNA sequence or gene expression data as input. I would like the authors to mention how their ncHMR detector outstands these available conventional tools. I recommend the authors to add a section in the introduction section supporting their statement.

*The published tools mentioned by the reviewer were designed for specific usages, and none of them can be applied to predict non-classical functions of HMRs. Among the published tools mentioned by the reviewer, HMCan and ChIPseqR were specially designed to identify genome-wide binding sites of HMs accurately, Epigram and DeepHistone can be used to predict binding sites of HMs based on sequence features and chromatin accessibility. As non-classical functions of HMRs are independent to their HM substrates / products, it’s necessary to focus on non-classical sites of HMRs lacking HM substrates / products rather than binding sites of HMs. Similarly, DeepChrome, EpiRegNet and Epidaurus were designed for revealing the regulatory functions of HMs on transcription regulation, which is fundamentally different to the non-classical functions of HMRs independently of their corresponding HMs. To our best knowledge, ncHMR detector is the first computational framework to predict the non-classical functions and cofactors of a given HMR systematically. We added a paragraph in Background to demonstrate the differences between our method and other methods mentioned by reviewer 1 and reviewer 2.*

b. The Abstract should be more descriptive, and the English language should be better phrased especially in regard to the grammar and sentence construction.

*According to the reviewer’s suggestion, we modified our abstract in the revised manuscript to ensure it is clear to understand.*

c. The link provided is a GitHub repository. Would it be possible for the authors to develop a web resource that allows users analyze their data sets. This would be key for biological researchers who do not have bioinformatics experience.

*According to the reviewer’s suggestion, we built a webserver (http://compbiozhanglab.org/ncHMR_detector/index.php) for users to analyze their datasets. The webserver also provides example files and usage instructions.*

d. When incorporating ChIP-Seq data sets. My major concern is why did the authors not consider querying the ENCODE database? I am certain the authors would have retrieved a larger data set in comparison to what they obtained primarily.

*We collected ChIP-seq datasets from Cistrome DB (http://cistrome.org/db/), an integrated data analysis and visualization portal for ChIP-seq data in human and mouse, which contains ENCODE datasets. Because ENCODE ChIP-seq datasets we used are also available in GEO, so we selected GEO accession number as sample ID in our pre-processed database for TFs in GitHub.*

*It should be noted that, we used a stringent quality control criterion (see Methods section for detail) to filter out some public ChIP-seq datasets to guarantee the quality of datasets used in the prediction framework.*

e. The authors mention that the ChIP-Seq data was "integrated" (page 14). The authors must provide more description as to how they performed the integration.

*According to the reviewer’s suggestion, we added more detailed description for data integration in Methods section in the revised manuscript.*

f. The authors use a lot of machine learning or deep neural network algorithms in their ncHMR detector. It is extremely important for the authors to explain they performed this analysis on the whole downloaded dataset or had they divided the data into training, validation and test sets in order to confer their model and check the accuracy.

*We used elastic net to predict the histone modification signals on HMR ChIP-seq peaks with the cobinding events of different potential cofactors. Elastic net is a regularized high-dimensional linear regression method with mixed L1 and L2 penalties and does not use a deep neural networks architecture. As a feature selection method, elastic net shrinks the effects of non-important factors to zero. Those features (cofactor candidates) selected by elastic net and further filtered by the refitted step are predicted as the cofactors for the non-classical functions. The tuning parameter in elastic net was selected by cross-validation, but the final prediction was performed on the full dataset. To show the reliability of our method, we confirmed our prediction with two published non-classical functions and provided experimental validation of a novel nonclassical function predicted by our method.*

g. Page 20. The following headings are missed in the methods sections. The headings include; statistical analysis (what packages they utilized), developing the ncHMR detector or data availability. It would also be good to have a web source heading that describes how the public resource was developed. I recommend the authors to include these sections.

*According to the reviewer’s suggestion, we added the following two parts in Methods section: 1) statistical analysis, 2) software and webserver implementation. Besides, we also added a paragraph for the availability of data and material in Declarations section.*

Minor comments:

a. Page 5 - ABL has not been expanded when used in the first instance.

*Abl is the abbreviation of LNCaP-abl, which is an androgen-independent prostate cancer cell line. The name of abl should be in lowercase. We mentioned the full name of abl in its first instance, and corrected it to lowercase in the revised manuscript.*

b. Page 6 - The link has already been mentioned in the introduction section. This should be removed.

*We removed the link in Page 6 in the revised manuscript.*

c. Page 6, 8, 9, 12 and 14 - The authors use terms such as "important factors", "other methods" and "dozens". These terms should not be used in research articles. It would be good to see the authors explain the results section in detail (for. e.g. what factors or methods were used or compared, how many "numbers" of non-classical functions were obtained).

*According to the reviewer’s suggestion, we modified those non-specific terms, and described our method and results clearly in the revised manuscript.*

d. Page 7 - Specificity using greedy forward selection and knock off values should be either represented separately as supplementary figures or a reference could be provided for the analysis results.

*The specificity using greedy forward selection and knock off are represented in Supplementary Fig. S2b-d.*

e. Page 10 - The authors use a term "CHROMO". It is not clear as to what the authors are referring *to.*

*CHROMO refers to CHRromatin Organization MOdifier domain, a protein domain of CBX7. We expanded it in its first instance in the revised manuscript.*

Reviewer #2:

This manuscript describes ncHMR detector that is designed to identify non-classical functions of HMRs. ncHMR detector relies on the significantly enriched co-occurrence of binding events of cofactors and the absence of classical substrates/products of the given HMR, and requires the availability of a large amount of ChIP-seq data for other factors in the same cell type. The proposed method was applied to the ChIP-seq data for GM12878, K562, hESCs and mESCs and in total 14 non-classical functions of HMRs were predicted. One of them, the non-classical function of CBX7 in mESCs was biologically validated. The manuscript could be of use to the field. My concerns are mainly about technical points elaborated on below.

1) Six of the top-10 HMRs (Fig. 3A) have broad marks (H3K27me3 and H3K9me3) as classical substrates/products. It may be inappropriate for such broad marks to focus on the small width (±1 kb flanking each ChIP-seq peak center of given HMR) because broad marks have much weaker S/N ratio than sharp peaks (e.g., H3K4me1) that makes difficult to distinguish whether each region is enriched or not. Does the proposed program assume the sharp and strong peaks of these marks? The discussion is necessary.

*We appreciate the reviewer’s comment, and we fully agree that ChIP-seq data of broad histone modifications have much weaker S/N ratio than those of sharp histone modifications. We acknowledge that ±1 kb window may be too narrow for broad histone modifications, such as H3K9me3, H3K27me3 and H3K36me3. In the revised manuscript, we modified our computational framework by using ±5 kb windows around HMR’s ChIP-seq peak centers to calculate the average signals for well-known broad histone modifications (H3K9me3, H3K27me3 and H3K36me3), while still using ±1 kb window for other histone modifications. We updated the prediction results in Fig. 3a and Supplementary Table S1 in the revised manuscript. The updated prediction results are largely consistent to our original predictions, indicating the robustness of our computational framework for HMRs with broad histone modification marks as substrates or products.*

2) Human and mouse ES cells are exceptional cases in which H3K27me3 is highly enriched around "poised promoter" TSSs. Because eight of top-10 HMRs (Fig. 3A) were from ES cells, the more results from other cell lines (e.g, GM12878, HeLa or HepG2) are required.

*We appreciate the reviewer’s comment. In the revised manuscript, according to the reviewer’s suggestion, we used data in six cell types / lines, including mESC, hESC, K562, GM12878, HeLa and HepG2. We predicted a non-classical function of CHD1 in GM12878, which ranks within top 10 (Fig. 3a). However, we did not predict any nonclassical functions of HMR in Hela and HepG2, which may due to too few qualified HMR ChIP-seq data available in both cell lines (4 HMRs in HeLa and 1 HMR in HepG2, shown in new Supplementary Fig. S4a).*

*From new Supplementary Fig. S4a, 76% HMR ChIP-seq data used in this study are from mESCs or hESCs, which can at least partially explain why 8 out of top 10 predicted non-classical functions of HMRs were from ES cells. In the future, with more qualified ChIP-seq data of HMRs available in other cell types / lines, we believe that more non-classical functions of HMRs can be predicted by using our computational framework.*

3) As related works for more general purposes, Gerstein et al. analyzed many TF ChIP-seq data and defined primary and local partners for each TF to identify contextspecific function of TFs (Gerstein et al, "Architecture of the human regulatory network derived from ENCODE data", Nature 2012). There are also many tools for "joint-peak analysis" (e.g. MultiGPS http://mahonylab.org/software/multigps/). While these are not designed for HMRs, they can be used. Please clarify the novelty of the proposed method against these existing studies.

*The published tools mentioned by the reviewer were designed for specific usages. The framework developed by Gerstein et al. can be used to define the partner factors with co-occurrence of binding events at specific subsets of a given HMR’s binding sites. But it cannot distinguish cofactors of classical functions and non-classical functions, because HMR can also perform classical functions by cooperating with cofactors. MultiGPS is a framework to analyze collections of multi-condition ChIP-seq data and characterize differential binding events between conditions for a given factor, which is not designed for context-dependent functions in the same condition. To our best knowledge, ncHMR detector is the first computational framework to predict the non-classical functions and cofactors of a given HMR systematically. We added a paragraph in Background section to demonstrate the fundamental differences between our method and other methods mentioned by reviewer 1 and reviewer 2.*

*We agree with the reviewer that some existing methods can be modified to perform the prediction of HMR’s non-classical functions. We compared the performance of ncHMR detector and four modified existing methods (MultiGPS + Jaccard index, edgeR + Jaccard index, DBChIP + Jaccard index, modified factor co-association analysis) by evaluating the robustness of cofactor identification, and ncHMR detector indeed outperformed those modified existing methods. We added a paragraph in Results, a supplementary Figure (Fig. S3) and a section in Methods to describe the comparison between the performance of our method and four modified existing methods.*

4) While the signals of HM (vector Y in Fig. 1d) are calculated quantitatively, TF peaks (matrix X in Fig. 1d) are evaluated binary (appear or not). Why does not the proposed method treat the signal of TF peaks quantitatively? That makes possible to evaluate not only the peak existence but also the quantitative change of shared peaks.

5) The proposed method simply uses peaks identified by MACS2 but it depends on the number of mapped reads. Therefore the sensitivity and specificity of the proposed method is dependent on the read number of the TF ChIP-seq data used. Taken together with my comment 3, quantitative value is desirable for TF peaks.

*As the reviewer’s comment 3 and 5 are not related, we assume the reviewer meant comment 4 and 5. Here, we combined comment 4 and 5 together to response.*

*We agreed that the binary overlap of two sets of ChIP-seq peaks does not represent the optimal approach when comparing peaks. However, in the default running mode of our computational framework, we still prefer to use binary values in matrix X with the following considerations. First, to use quantitative values in matrix X, it will take much longer time to parse bigwig files of TF ChIP-seq datasets than using binary values. For example, when running data in mESCs, it will take more than 20 minutes by using quantitative values, compared to about 2 minutes by using binary values. Second, according to the reviewer’s suggestion, we added a running mode (using quantitative values in matrix X) in our computational framework. As shown in Fig. R1, the top predicted non-classical functions of HMRs in both modes are quite similar, and the predicted non-classical cofactors in both modes are largely consistent. Considering the dramatic difference of running speed and the consistence of the results between two modes, we prefer to keep using binary values in matrix X as the default running mode. Nevertheless, advanced users can choose another running mode (using quantitative values in matrix X) when running our software released in GitHub.*

6) Each HMR can have two or more non-classical functions other than the classic function, while the proposed method may not be able to distinguish them. Please add the discussion for this point.

*We agree with the reviewer that each HMR can have multiple non-classical functions cooperating with distinct cofactors. Our framework can report different subsets of nonclassical sites for different cofactors, but it does not report whether different cofactors represent distinct non-classical functions. The users can further analyze the lists of non-classical sites for different cofactors to classify the potential multiple non-classical functions of a given HMR. According to the reviewer’s suggestion, we added the discussion for this point in Discussion section in the revised manuscript.*

7) In Fig 4a, some of CBX7 peaks colocalized with H3K27me3, H3K9me3 and NANOG. Does this result suggest NANOG also functions in the classical CBX7 peaks?

*As the reviewer mentioned, a small fraction (2.4%) of CBX7 peaks colocalize with H3K27me3 and NANOG. To investigate whether NANOG also functions in those CBX7 peaks, we performed the genomic distribution of those peaks. As shown in Fig. R2, 68% of those peaks are located in intergenic regions, which is quite different to that of all classical CBX7 peaks (20%, shown in Fig. 4b). We further performed the Gene Ontology analysis for the nearest genes of CBX7 peaks colocalized with H3K27me3 and NANOG, but none of GO terms are enriched. Although we cannot exclude the possibility that NANOG can function in a small fraction of CBX7 classical peaks, such discussion is beyond the scope of this study. We can explore the possibility in the future studies.*

8) Though the evaluation of the non-classical function of CBX7 confirmed the effectiveness of the proposed method, the other predicted functions (Fig. 3a) also need to be discussed referring the existing biological studies. For example, CTCF was identified as a cofactor of KMT2B, but CTCF itself has many cofactors such as cohesin (Wendt. Nature 2008). Why were not the cofactors of CTCF identified in this analysis whereas the cohesin ChIP-seq data for mESC is available?

*We appreciate the reviewer’s comment. In our original manuscript, we did not collect ChIP-seq data of cohesin (RAD21 and SMC3). In our revised manuscript, we added RAD21 and SMC3 ChIP-seq data, and updated the predicted results. As shown in updated Fig. 3a, RAD21, CTCF and SMC3 were all predicted to be non-classical cofactors of KMT2B.*

9) In some cases, non-classical sites and classical sites may be functionally correlated (e.g. enhancer and promoter sites for same genes). Do the authors have comments about the way to distinguish whether identified non-classical function is correlated with the classical one? One possibility may be the averaged distance between the sites.

*We appreciate the reviewer’s suggestion. In the revised manuscript, we investigated whether some HMRs’ non-classical functions are correlated with their classical functions. For each predicted HMR with non-classical function, we first compared the average distances of non-classical sites to their nearest classical sites and to the nearest other non-classical sites. Among 12 predicted HMRs with non-classical functions, KDM5A, RBBP5 and WDR5 showed significantly closer distance between their non-classical sites and classical sites (updated Supplementary Fig. S4d). We further investigated their chromatin interaction frequencies using public Hi-C data, and we observed significantly higher interaction frequencies between non-classical sites and their nearest classical sites for KDM5A, RBBP5 and WDR5 (updated Supplementary Fig. S4e). The results suggested the non-classical functions of KDM5A, RBBP5 and WDR5 might be correlated with their classical functions via chromatin looping. The above analysis was added in the revised manuscript.*

10) In addition to Fig. S4e, the RT-qPCR result for the expression of NANOG in Ezh2 +/+ and Ezh2 -/- is necessary (same as Fig. 4C).

*According to the reviewer’s suggestion, we performed additional experiments to compare the expression of NANOG in Ezh2 +/+ and Ezh2 -/- mESCs. Consistent with our expectation, NANOG expression remain unaffected by Ezh2 deletion. The RTqPCR result was added as Supplementary Fig. S5e in the revised manuscript.*

11) The main manuscript is a bit redundant. Especially, several sentences in Conclusion were very similar to text in Discussion.

*We edited our manuscript and removed the redundant part.*

Minor:

1) I strongly recommend updating the proposed method from Pyhon2 to Python3 because Python2 will retire in the next year.

*According the reviewer’s suggestion, we provided both Python 2 and Python 3 versions of our computational framework in GitHub (*[*https://github.com/TongjiZhanglab/ncHMR_detector*](https://github.com/TongjiZhanglab/ncHMR_detector)*).*

2) Please add the time of experiments and the p-value to show the statistical significance for Fig. 4c and Fig. S4c.

*We added the time of experiments in figure legends and the statistical significance in revised Fig. 4c and Supplementary Fig. S5c.*

Reviewer #3:

This is an interesting study with a novel tool, ncHMR detector, which is the first computational framework to predict the non-classical functions and cofactors of a given HMR systematically, based on ChIP-seq data integration. It is an important question to identify the non-classical functions of HMRs due to its essential involvement in a variety of cellular processes. However, there is no framework designed for identifying such functions systematically except few cases reported in the literature. Generally speaking, this is a nice study except few questions listed below:

Major:

1. To some extent, it is hard to evaluate the prediction accuracy of the tool though the author has done few confirmations. What is the FDR for ncHMR detector? Will it be affected by your choice of R cutoff?

2. What is the rationale to choose R<0.1?

*As the first two major issues of the reviewer actually pointed to the same question, we combined them together to response. We agree that it is hard to evaluate the FDR for our computational framework. Here, we performed a robustness analysis by comparing the predicted cofactors with different R2 cutoffs (as shown in Fig. R3). We found that the results were quite stable at the given range of R2 cutoffs (from 0.01 to 0.2, as shown in Fig. R3). By default, we set a stringent R2 cutoffs (0.1) with an empirical p-value cutoff (0.001). Users can also change R2 cutoffs by setting the parameter --R-squared. The higher stringent R2 cutoffs lead to less candidates. Our computational framework reported the candidates ranking from highest R2 to the lowest. Users can go through all the candidates to focus on those they are interested in. We added the description for R2 cutoff in Methods section in the revised manuscript.*

3. In the second step, what cutoff was used to select significant factors with feature selection by elastic net or Lasso?

*In the factor selection step (second step), we used standard elastic net to predict histone modification signals on HMR peaks with the cobinding events with different potential co-factors. By default, we set α=0.5 to control the relative weighting of Lasso and Ridge. Besides, we used the value of lambda.1se as the selected value for λ in order to provide a simpler model with comparable error to the best model. The cofactor candidates with non-zero coefficient are selected as significant factors in the factor selection step and used as the input of the following steps. Users can change the default values by setting the parameters --Alpha and --LambdaChoice. We added the above details in Methods section in the revised manuscript.*

Minor: In fig. 4, Cbx7-knockout (KO) has less effect on Nanog expression compared with Cbx7-knockdown, confirmed by both Western blot (fig. 4d) and immune-staining (fig. 4f). Any explanation? Cbx7-KO should have more profound effect on Nanog expression compared with partial knockdown of Cbx7.

*The western blot assay showed that the expression level of NANOG was decreased in Cbx7-knockout or Cbx7-knockdown cells to a similar extent compared with wild Scr. We agree that immunofluorescence figure (Fig. 4f) could be misleading due to only a few cells represented. Here, we measured the fluorescence intensity of NANOG in about 300 nuclei in each condition. The statistics showed that there was no significant difference in NANOG intensity between Cbx7-knockdown and Cbx7-knockout cells. The figure of NANOG fluorescence intensity was added as Fig. 4g.*

**Second round of review**

**Reviewer 1**

Not of sufficient advance so recommend a more specialized journal.

**Reviewer 2**

I am satisfied with the authors' response and the revision to the manuscript but one minor question:

1)     Broad histone marks especially H3K9me3 are typically enriched broadly (more than 100 kbp) while in some special cases they are enriched as "sharp peaks" mainly around TSSs. The proposed method seems to consider the latter case (because it focuses on the at most 10 kbp region centered by HMR peaks). I'd like to know whether such H3K9me3 enrichment detected by the proposed method (e.g., Fig. 3a K562 and Fig. 4a) can be identified by typical peak-calling method such as MACS2 considering that the HM enrichment is not necessarily statistically significant because the signals of HM are calculated quantitatively. This point is important for the histone modification analysis field.

**Reviewer 3**

The authors have made significant efforts to improve the manuscript. It is expected to obtain enthusiastic feedbacks from the readers.

**Authors Response**

**Point-by-point responses to the reviewers’ comments:**

Reviewer #2:

I am satisfied with the authors' response and the revision to the manuscript but one minor question: 1) Broad histone marks especially H3K9me3 are typically enriched broadly (more than 100 kbp) while in some special cases they are enriched as "sharp peaks" mainly around TSSs. The proposed method seems to consider the latter case (because it focuses on the at most 10 kbp region centered by HMR peaks). I'd like to know whether such H3K9me3 enrichment detected by the proposed method (e.g., Fig. 3a K562 and Fig. 4a) can be identified by typical peak-calling method such as MACS2 considering that the HM enrichment is not necessarily statistically significant because the signals of HM are calculated quantitatively. This point is important for the histone modification analysis field.

*In this study, we focused on ± 5 kb regions around HMR’s peak centers when its substrate/product was well-known broad histone modification (such as H3K9me3). We avoided to use a very broad window (such as 100 kb), because our approach focused on the histone modification signals around HMR’s ChIP-seq peaks (such as SETDB1’s peaks), which are usually not broad.*

*According to the reviewer’s comment, we analyzed SETDB1 and H3K9me3 ChIP-seq data in K562 to examine whether H3K9me3 enrichment classified in our study can be identified by typical peak-calling method MACS2. As shown in Figure R1, 91.7% identified SETDB1 classical sites in this study (i.e. with strong H3K9me3 signals) were identified as H3K9me3 peaks by MACS2 (broad peak mode), while the percentage is only 15.6% for identified SETDB1 non-classical sites in this study (i.e. with weak H3K9me3 signals). Besides MACS2, we also tried ChromHMM, an additional method for histone modification ChIP-seq analysis, and we observed a similar result (Figure R1). Our analysis indicated that the H3K9me3 enrichment classification in this study is quite consistent to peak-calling results.*
